# Obesity, adipokines, and C-peptide are associated with distinct plasma phospholipid profiles in adult males, an untargeted lipidomic approach

**DOI:** 10.1038/s41598-017-05785-0

**Published:** 2017-07-24

**Authors:** C. Austin Pickens, Ana I. Vazquez, A. Daniel Jones, Jenifer I. Fenton

**Affiliations:** 1 Department of Food Science and Human Nutrition, Michigan State University, 469 Wilson Road, East Lansing, MI 48824 USA; 2 Department of Epidemiology and Biostatistics, Michigan State University, 909 Fee Road East Lansing, MI 48824 USA; 3 Department of Biochemistry and Molecular Biology, Michigan State University, 603 Wilson Road, East Lansing, MI 48824 USA; 4 Department of Chemistry, Michigan State University, 578 S Shaw Lane, East Lansing, MI 48824 USA

## Abstract

Obesity is associated with dysregulated lipid metabolism and adipokine secretion. Our group has previously reported obesity and adipokines are associated with % total fatty acid (FA) differences in plasma phospholipids. The objective of our current study was to identify in which complex lipid species (i.e., phosphatidylcholine, sphingolipids, etc) these FA differences occur. Plasma lipidomic profiling (n = 126, >95% Caucasian, 48–65 years) was performed using chromatographic separation and high resolution tandem mass spectrometry. The responses used in the statistical analyses were body mass index (BMI), waist circumference (WC), serum adipokines, cytokines, and a glycemic marker. High-dimensional statistical analyses were performed, all models were adjusted for age and smoking, and p-values were adjusted for false discovery. In Bayesian models, the lipidomic profiles (over 1,700 lipids) accounted for >60% of the inter-individual variation of BMI, WC, and leptin in our population. Across statistical analyses, we report 51 individual plasma lipids were significantly associated with obesity. Obesity was inversely associated lysophospholipids and ether linked phosphatidylcholines. In addition, we identify several unreported lipids associated with obesity that are not present in lipid databases. Taken together, these results provide new insights into the underlying biology associated with obesity and reveal new potential pathways for therapeutic targeting.

## Introduction

Obesity is associated with insulin resistance, oxidative stress, chronic low-grade inflammation, and dysregulated secretion of adipose-derived cytokines (i.e., adipokines)^1^. Obesity is also associated with altered lipid metabolism (reviewed in detail ref. 2) and many lipids function as signaling molecules involved in inflammation pathways. The chronic low-grade inflammation in obesity can alter insulin receptor downstream signaling leading to insulin resistance (reviewed in detail ref. 3), and insulin resistance can affect lipid storage and lipid metabolism^4^.

The human plasma lipidome is comprised of lipid species such as glycerolipids, fatty acids (FAs), cholesterol esters, and phospholipids (PLs) including the glycerol-based diacylphospholipid and lysophospholipid (LPL), and the sphingosine-based sphingolipids^5^. PL contain esterified fatty acids (FAs), for instance, there are two esterified FAs in diacylphospholipids such as: phosphatidylcholine (PC), phosphatidylethanolamine (PE), phosphatidylserine (PS), phosphatidylglycerol (PG), and phosphatidylinositol (PI), and there is one esterified FA in their respective LPLs: LPC, LPE, LPS, LPG, and LPI. FAs are endogenously synthesized except for the essential polyunsaturated FA (PUFAs) linoleic acid (LA), a C18:2^9Z,12Z^ omega-6 (ω-6), and alpha-linolenic acid (ALA), a C18:3^9Z,12Z,15Z^ omega-3 (ω-3). Obesity is associated with increased elongation, desaturation, and oxygenation of FAs, which can lead to higher levels of LA-derived long chain PUFAs such as arachidonic acid (ARA), a C20:4^5Z,8Z,11Z,14Z^ ω-6^2, 6^, and ARA-derived proinflammatory signaling lipids^7^. Despite FA metabolites being directly involved in inflammatory signaling, the metabolism of PLs are also involved in activating signal transducing pathways associated with inflammation regulation [reviewed in detail ref. 8].

The metabolism of diacylphospholipids results in the production of LPs, which are generated by lecithin:cholesterol acyltransferase (LCAT)^9^, and phospholipase A_1_ (PLA_1_) and A_2_ (PLA_2_)^10^. LPLs such as LPCs are ligands for G-protein coupled receptors (reviewed in detail ref. 11) and can exert either pro-inflammatory^12, 13^ or anti-inflammatory responses depending on the FA chain esterified^14^. Several studies report obesity is inversely associated with LPLs. For instance, overweight and obese individuals have lower LPC(18:1) and (18:2)^15^, and Rauschert *et al*. reported WC is inversely associated with LPC(18:1) and (18:2)^16^. Higher levels of LPC(18:2) were independently associated with a decreased risk of type-2 diabetes in a European Prospective Investigation into Cancer and Nutrition (EPIC)-Potsdam study^17^. In a recent randomized control trial, supplementation with ω-3 PUFAs increased ω-3s in LPLs and lowered ω-6s in LPLs, but overall LPLs were still lower in obese compared with normal and overweight individuals^18^. Some studies suggest that increases in some LPLs such as LPC(18:0) are indicators of high fat diet induced obesity^19^, and in obesity resistant mice, HFD increases plasma LPCs in particular LPC(18:0), (18:1), (18:2), (22:6)^20^. The decrease in plasma LPLs may result from altered lipid metabolism in the liver^21^, since LPLs changes are associated with fatty liver^22, 23^. Although there are conflicting reports on whether specific plasma LPLs increase^15, 21, 24^ or decrease^8, 15, 25^ in obesity, there is a clear link between LPLs and obesity-associated comorbidities.

Our group has previously reported that plasma PL FA levels were associated with obesity^26^, adipokines, and the glycemic marker C-peptide^27^. In these studies, the plasma PL FA measurements were obtained using a FA methyl ester (FAME) analysis and by analyzing PL FAs as % of total FAs. PL FAME analyses involve hydrolyzing FAs from their parent PL (i.e., glycerol-based PLs, sphingolipids, etc), meaning the PLs class (i.e., LPLs) associated with the FA differences cannot be determined. We hypothesized that our previously reported plasma PL FA differences associated with obesity^26^ and cytokines^27^ occur in specific PLs. In our current study, we analyzed plasma collected from the same patients at the same time point as the plasma analyzed in our previous FAME studies^26, 27^, to determine these specific PLs with differing FAs. We investigated associations between the traits obesity, adipokines, proinflammatory cytokines, and C-peptide, and intact plasma PLs by using negative-mode high-resolution tandem mass spectrometry (MS/MS) to 1) identify specific plasma PL species with FA differences associated with the traits, and 2) assess the contribution of the entire plasma lipidome on the variation of traits using multidimensional statistical methods.

## Results

### Participant characteristics

Median and range values of age, smoking, anthropometric, and serum adipokines, glycemic, and inflammatory markers of the overall population (n = 126) and separated by BMI categories are presented in Table 1. In brief, lean and obese participants were older than overweight participants. In the obese group, 19 patients had BMI > 35 and only 6 patients had BMI > 40. Both BMI and WC increased with increasing BMI category. Serum leptin and C-peptide significantly increased across BMI category, and serum adiponectin was decreased in obese participants compared to lean and overweight participants. Serum TNF-α and IP-10 were significantly elevated in obese participants compared to overweight and lean participants, while MCP-1 was only elevated in obese participants compared to lean.

**Table 1:** Median [range] of the participant characteristics, in the sample, and divided by BMI category.

Parameter	Overall n = 126^a^	Lean n = 28^a^	Overweight n = 46^a^	Obese n = 52^a^	BH FDR p-value^b^
Age (years)	58 [48–65]	58 [50–65]^A^	54 [48–65]^B^	59 [50–65]^A^	p < 0.005
Ever Smoked (% total)	31	25	24	27	—
BMI (kg/m^2^)	29.1 [19.2–45.6]	23.6 [19.2–25.0]^A^	28.1 [25.4–29.9]^B^	33.7 [30.0–45.6]^C^	p < 0.0001
WC (cm)	104.8 [75.6–146.1]	85.7 [76.2–101.6]^A^	101.6 [82.5–113.7]^B^	119.4 [75.6–146.1]^C^	p < 0.0001
Leptin (ng/mL)	6.1 [0.3–49.5]	2.1 [0.3–6.0]^A^	5.2 [0.7–10.2]^B^	13.7 [3.8–49.5]^C^	p < 0.0001
Total Adiponectin (μg/mL)	4.2 [1.0–13.2]	5.4 [1.9–13.2]^A^	4.8 [2.2–8.5]^A^	3.7 [1.0–8.5]^B^	p < 0.005
C-peptide (ng/mL)	2.4 [0.7–9.9]	1.4 [0.7–5.1]^A^	2.1 [0.9–8.1]^B^	3.3 [1.0–9.9]^C^	p < 0.0001
IL-6 (pg/mL)	1.6 [0.0–137]	0.2 [0.0–40.9]^A^	1.0 [0.0–96.6]^A^	3.4 [0.0–137]^A^	—
TNF-α (pg/mL)	7.4 [1.6–67.1]	5.7 [2.8–26.2]^A^	7.3 [1.6–24.1]^A^	8.6 [1.6–67.1]^B^	p < 0.0005
IP-10 (pg/mL)	306 [120–1192]	257.5 [120–557]^A^	271 [145–1029]^A^	378 [147–1192]^B^	p < 0.0005
MCP-1 (pg/mL)	483 [85–1018]	428 [218–875]^A^	488 [285–1018]^AB^	511 [85–922]^B^	p < 0.05

Body mass index (BMI), waist circumference (WC), tumor necrosis factor-alpha (TNF- α), interferon gamma-induced protein-10 (IP-10), interleukin-6 (IL-6), monocyte chemoattractant protein-1 (MCP-1).

^a^Values expressed as median [range]. Cytokines quantified from serum.

^b^Kruskall Wallis one-way ANOVA was conducted across BMI categories along with Dunn’s test for multiple comparison. The superscripted letters A, AB, B, and C represent the multiple comparison test for each parameter for the lean, overweight, and obese categories. P-values were adjusted using Benjamini-Hochberg false discovery rate (BH FDR) correction.

### Lipidomic profiles explain a large proportion of inter-individual variation of obese traits

The lipidomic profiles accounted for an important proportion of the inter-individual variation of all responses (Table 2). For several traits (i.e., BMI, WC, and leptin) lipidomic profiles accounted for over 60% of the variation in the samples (Table 2). Lipidomic profiles accounted for roughly 40% of the variation in total-adiponectin and C-peptide concentrations. For the inflammatory cytokines IL-6, TNF-α, IP-10, and MCP-1, lipidomic profiles accounted for about 25% of variation in the samples. The 95% confidence intervals for most responses was roughly 10–20% for each respective trait (Table 2).

**Table 2:** Percent of the inter-individual differences in response variables that can be attributed to lipidome profiles, posterior mean [95% Confidence region].

Parameter	${{\boldsymbol{\sigma }}}_{{\boldsymbol{\mu }}}^{2}/{{\boldsymbol{\sigma }}}_{{\boldsymbol{\mu }}}^{2}+{{\boldsymbol{\sigma }}}_{{\boldsymbol{\varepsilon }}}^{2}{\boldsymbol{ \% }}$ [95% CI]
BMI	64% ± [38%, 84%]
WC	69% ± [46%, 85%]
Leptin	76% ± [56%, 90%]
Total Adiponectin	42% ± [21%, 66%]
C-peptide	48% ± [23%, 73%]
IL-6	23% ± [10%, 43%]
TNF- α	28% ± [12%, 54%]
IP-10	23% ± [10%, 43%]
MCP-1	24% ± [11%, 45%]

Body mass index (BMI), waist circumference (WC), tumor necrosis factor-alpha (TNF- α), interferon gamma-induced protein-10 (IP-10), interleukin-6 (IL-6), monocyte chemoattractant protein-1 (MCP-1).

### Phospholipid classes and individual phospholipids and are associated with obese traits

**Figure 1:**
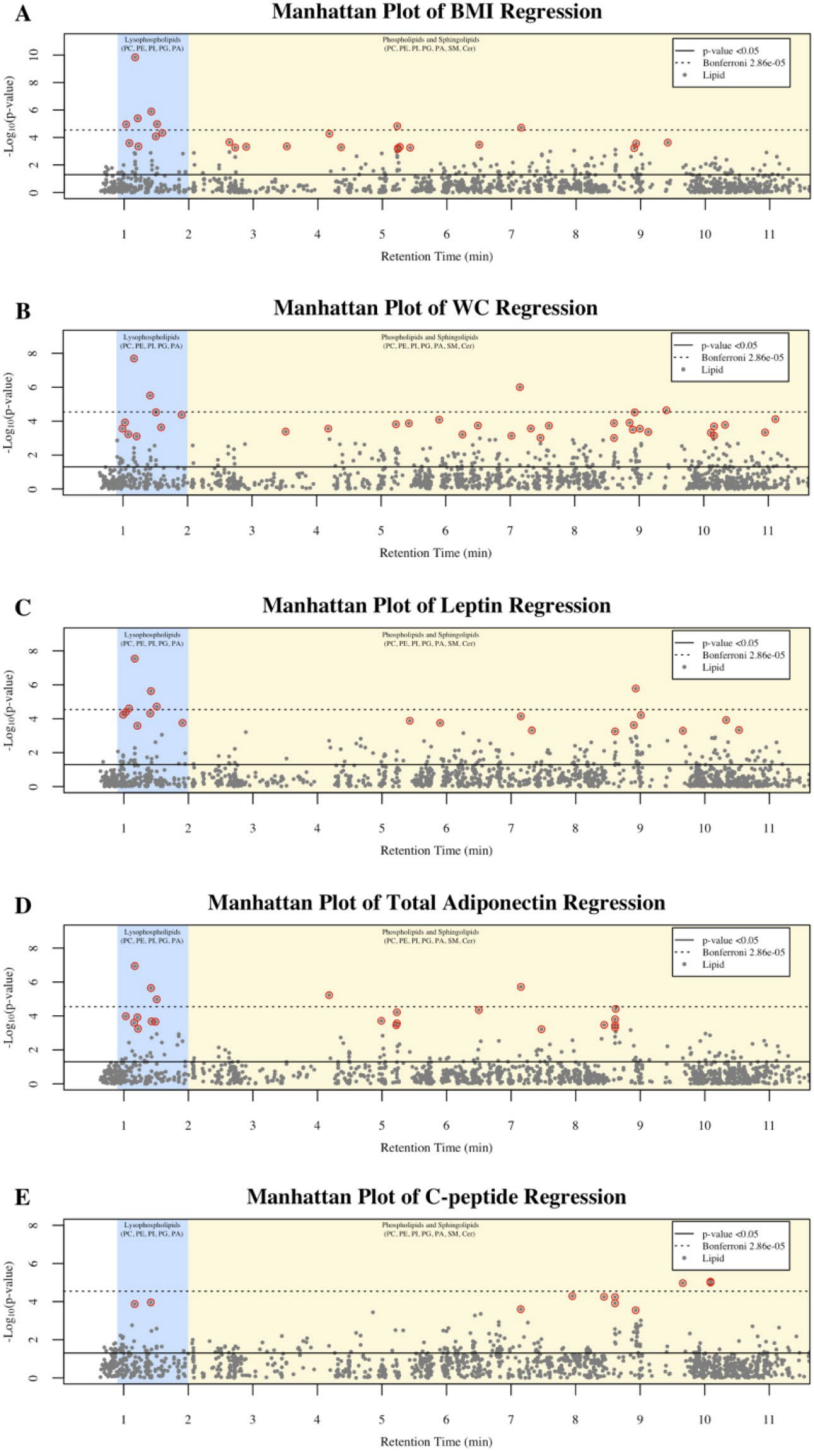
Manhattan plots of the –log10(p-value) for the traits: (**A**) BMI, (**B**) WC, (**C**) Leptin, (**D**) Total Adiponectin, and (**E**) C-peptide. Single lipid regression models defined as: Trait_*i*_ = Age_*i*_ + Smoking_*i*_ + Lipid_j_ where *i* = (1, …, 126) and *j* = (1, …, 1745). Each y-axis represents −log10(p-value) for each respective model, and each x-axis represent the retention time of the metabolite in minutes. Plasma lipid Benjamini-Hochberg false discovery corrected p-values < 0.05 are circled in red. Body mass index (BMI), waist circumference (WC), tumor necrosis factor-alpha (TNF- α), interferon gamma-induced protein-10 (IP-10), interleukin-6 (IL-6), monocyte chemoattractant protein-1 (MCP-1).

Results of age and smoking adjusted single lipid regressions are outlined in Fig. 1 for the traits BMI, WC, leptin, total adiponectin, and C-peptide, and for each trait the primary id, estimated effects, and p-values of lipids with BH FDR p-values < 0.05 are displayed in Supplementary Tables 2, 3, 4, 5 and 6, respectively. Since no lipids were significantly associated with any response after 11.5 mins, the Manhattan plots were created to span from 0.5 (i.e., the start of MS/MS data collection) to 11.5 mins for clarity. In BMI regressions, 25 plasma lipids had BH FDR p-values < 0.05 and LPLs accounted for over 70% of lipids with Bonferroni p-values < 0.05 (Fig. 1A). WC was significantly associated with 35 lipids below the BH FDR (Fig. 1B). Leptin was significantly associated with 20 plasma lipids below the BH FDR (Fig. 1C). In total adiponectin regressions there were 22 lipids below the BH FDR (Fig. 1D). For WC, leptin, and total adiponectin, over 50% of lipids with Bonferroni p-values < 0.05 were LPLs (Fig. 1B,C and D, and Supplementary Tables 3, 4 and 5). C-peptide was significantly associated with 11 plasma lipids below the BH FDR (Fig. 1E). Neither IL-6, IP-10, or MCP-1 had any lipid significant below the BH FDR (data not shown).

**Figure 2:**
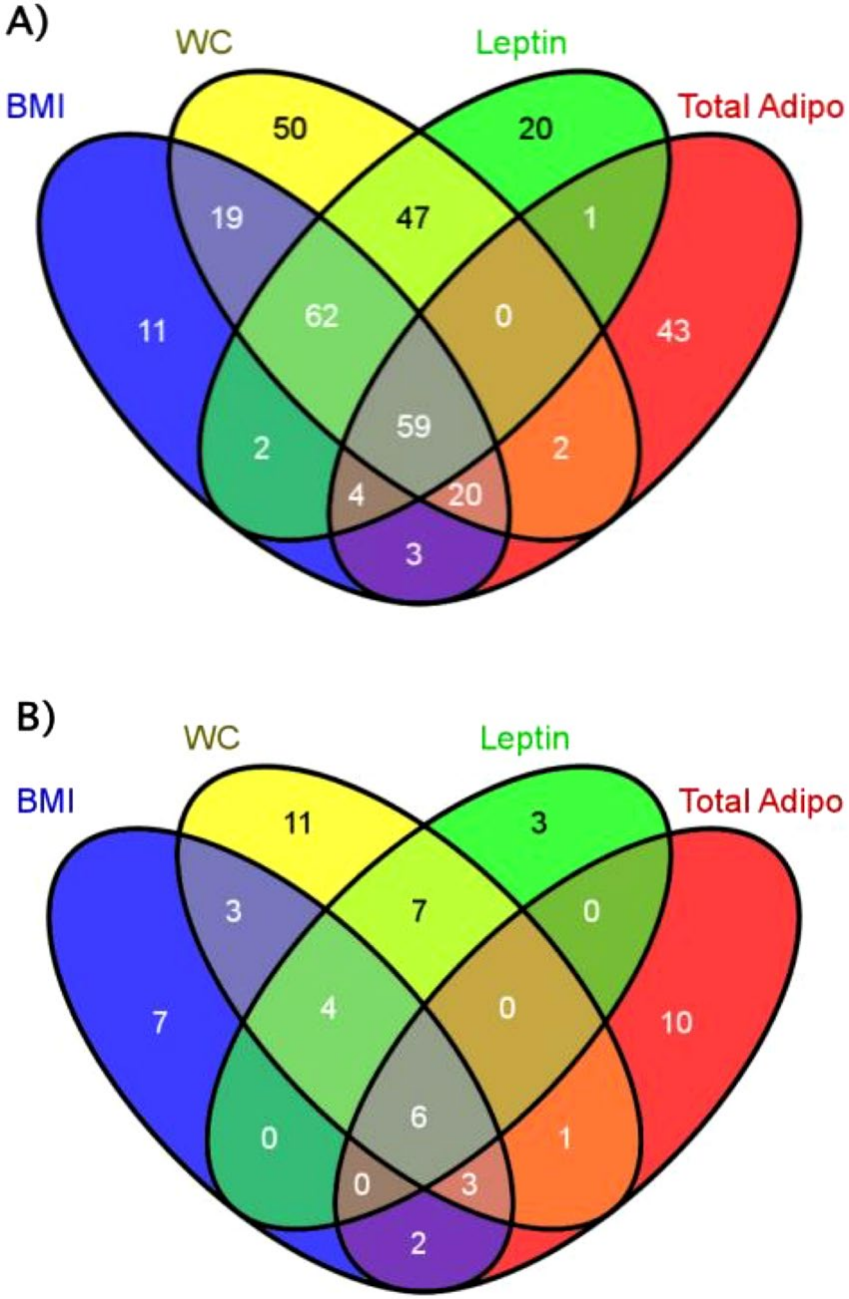
Venn diagrams displaying the relationship of lipids significantly associated between the traits body mass index (BMI), waist circumference (WC), leptin, and total adiponectin (Total Adipo). (**A**) Relationship of lipids with p-values < 0.05 for the traits. (**B**) Relationship of lipids with Benjamini-Hochberg p-values < 0.05.

Next, lipids significant across several traits from single lipid regression were inputted into the software Venny^28^. This was performed to illustrate the number of significant lipids with un-adjusted p-values and the number of significant lipids with BH FDR adjusted p-values, from each trait using Venn diagrams. Since Venny creates a Venn diagram for up to 4 traits, the anthropometrics BMI and WC, and the adipokines leptin and total adiponectin were selected for the illustration. Relationships between the traits and lipids with p-values < 0.05 are presented in Fig. 2A. There were numerous lipids with p-values < 0.05 associated with traits (Figs 1 and 2A), for instance: 180 lipids with BMI, 259 lipids with WC, 195 lipids with leptin, and 132 lipids with total adiponectin. In total, there were 59 lipids associated between the four traits (Fig. 2A). A Venn diagram of only lipids with BH FDR p-values < 0.05 for the traits are presented in Fig. 2B. There were 6 lipids with BH FDR p-values < 0.05 associated between the four traits (Fig. 2B), and the primary ids were: X1.17_564.3289, X1.42_566.3497, X1.21_476.2768, X1.51_592.3513, X1.03_562.3132, and X7.15_786.5626. A complete list of lipids significant in at least 2 or more single marker regression models are outlined in Table 3.

**Table 3:** Identification of plasma lipid biomarkers.

Primary ID	Common name	Models where significant
X0.99_586.3136	LPC(20:5)	WC, leptin, Pc4
X1.03_562.3132	LPC(18:3)	BMI, WC, leptin, total adipo
X1.08_612.3280	LPC(22:6)	BMI, WC, leptin
X1.17_564.3289	LPC(18:2)	BMI, WC, leptin, total adipo, C-peptide
X1.21_476.2768	LPE(18:2)	BMI, WC, leptin, total adipo
X1.22_544.2648	LPS(20:4)	BMI, total adipo
X1.35_669.3989	Unknown	SVD
X1.42_566.3497	LPC(18:1)	BMI, WC, leptin, total adipo, C-peptide
X1.49_478.2927	LPE(18:1)	BMI, total adipo
X1.51_592.3513	LPC(20:2)	BMI, WC, leptin, total adipo, SVD
X1.59_554.3446	LPC(17:0)	BMI, WC
X1.91_594.3757	LPC(20:1)	WC, leptin
X2.65_255.2331	Palmitic acid	Pc4
X3.52_421.3076	TLTI	BMI, WC, SVD
X4.18_794.5050	Unknown	BMI, WC, total adipo
X4.37_881.5140	PI(16:0_22:6)	Pc4
X5.23_826.5592	PC(18:2_18:2)	BMI, WC, total adipo
X5.43_1068.6601	Unknown	BMI, WC, leptin
X5.90_810.5600	PC(O-16:0_20:5)	WC, leptin
X6.50_828.5729	PC(18:1_18:2)	BMI, WC, total adipo
X7.15_786.5626	PC(O-16:1_18:2)	BMI, WC, leptin, total adipo, C-peptide
X7.32_864.6056	PC(O-18:1_22:5)	WC, leptin
X7.47_788.5783	PC(O-16:0_18:2)	WC, total adipo
X8.17_830.6155	PC(18:0_18:2)	SVD
X8.23_950.6408	TLTI	Pc4
X8.24_880.6145	PC(20:1_20:4)	SVD
X8.44_716.5227	PE(16:0_18:1)	total adipo, C-peptide
X8.61_1038.473	TLTI	WC, C-peptide
X8.61_766.5363	PE(18:0_20:4)	WC, leptin, total adipo, C-peptide
X8.67_864.6048	PC(O-18:0_22:6)	Pc4
X8.90_880.6027	PC(18:0_22:5)	BMI, WC, leptin
X8.93_856.6036	PC(18:0_20:3)	BMI, WC, leptin, C-peptide
X9.01_1128.5388	TLTI	WC, leptin
X9.03_842.5446	TLTI	Pc4
X9.42_814.5927	PC(O-18:1_18:2)	BMI, WC
X9.45_1261.8069	TLTI	Pc4
X9.66_768.5494	PE(18:0_20:3)	leptin, C-peptide
X9.96_1034.5762	TLTI	Pc4
X10.11_1381.848	Unknown	WC, Pc4
X10.19_802.5695	TLTI	Pc4
X10.33_871.6904	SM(d19:1/24:1)	WC, leptin, Pc4
X11.20_1012.7670	TLTI	Pc4
X12.14_850.7682	TLTI	SVD
X12.43_934.7918	TLTI	SVD
X13.07_988.794	TLTI	Pc4
X13.10_920.7410	TLTI	Pc4
X13.11_919.7343	TLTI	Pc4
X14.31_881.7973	TLTI	Pc4
X14.33_869.3325	TLTI	Pc4
X14.35_1062.754	TLTI	Pc4
X14.38_880.7349	TLTI	Pc4

The structural of identification of plasma lipids was performed based if *m/z*: was significant in two or more models from single marker regression analyses of responses, or had a radii ≥ 0.15 in singular value decomposition analysis, or was one of the top ten lipids positively and inversely associated with principal component 4. Plasma lipids meeting this criterion that were too low to identify (TLTI) are listed. Plasma lipid common names determined by lipid structure identification using Lipid Maps and Human Metabolome databases, and manual confirmation of mass spectra. The “_” between fatty acids in glycerophospholipid structures is used, since the position at the sn1 and sn2 position cannot be determined. Plasma lipids listed as unknown did not match a lipid library database search or exhibit a fragmentation pattern indicative of phospholipids in ms/ms analyses. Spectra of unknowns are listed in Supplementary Table 9. Body mass index (BMI), waist circumference (WC), total adiponection (total adipo), principal component 4 (Pc4) and singular value decomposition (SVD).

### Biplot

**Figure 3:**
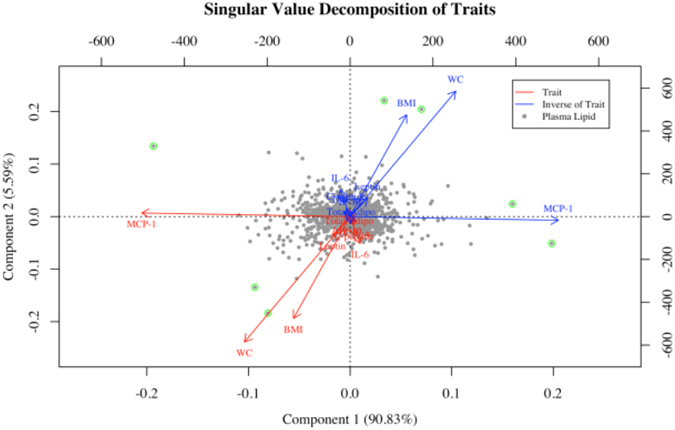
Singular value decomposition modeled on the 1,745 regression beta coefficients from plasma lipids in age and smoking regression for each respective parameter. () represent the plasma lipid loading score into vectors 1 and 2. Plasma lipids are highlighted if radius >0.15. Parameters are listed in red text with red arrows and the inverse of the parameters are listed in blue text with blue arrows. The score of MCP-1 in vector 1 is scaled by a factor 25 (i.e., original score >12,000). Body mass index (BMI), waist circumference (WC), tumor necrosis factor-alpha (TNF- α), interferon gamma-induced protein-10 (IP-10), interleukin-6 (IL-6), monocyte chemoattractant protein-1 (MCP-1).

A biplot analysis (Fig. 3) was derived from the 1,745 beta coefficients from each respective lipid in single marker regressions and the 9 traits (i.e., BMI, WC, leptin, total adiponectin, C-peptide, IL-6, IP-10, and MCP-1). Data were multidimensional, thus a biplot was used to extend the bivariate scatter plot. Biplots allow displaying information on samples and variables simultaneously. Here, a matrix with the lipid effects was built with samples in the rows (beta coefficients of the 1,745 samples) and 9 different lipid traits in the columns (i.e., BMI, WC, leptin, total adiponectin, C-peptide, IL-6, IP-10, and MCP-1). That matrix was decomposed in three using SVD, and in the biplot presented in Fig. 3, samples are displayed as points while traits are displayed as vectors. The vectors 1 and 2 from the SVD derived from the plasma lipid estimated effects, are represented with dots, and the loadings of the traits are represented with red arrows (in general, leftmost and bottom axes), and the inverse loadings of the traits are represented with blue arrows (in general, uppermost and right axes). Vectors 1 and 2 accounted for 96% of the variation in the lipidomics profiles. The orthogonal projection of the dots in the vectors are an approximation of the effect of each lipid for that trait. For instance, X12.43_934.7918 contributes to higher MCP-1 concentrations, while X8.17_830.6155 and 12.14_850.7682 both contribute to lower MCP-1 concentrations. The lipids X8.24_880.6145 and 3.52_421.3076 contributed to higher BMI, WC, and MCP-1 concentrations, and the lipids X1.51_592.3513 and X1.35_669.3989 contributed to lower BMI, WC, and MCP-1 concentrations. In addition, the score of MCP-1 in the first vector was scaled by a factor 25 (i.e., original score >12,000) to display it within the range of the other traits in the figure. The traits IL-6, leptin, total adiponectin, C-peptide, and IP-10 did not significantly load into vectors 1 and 2.

### Different BMIs display different lipidomic profiles

**Figure 4:**
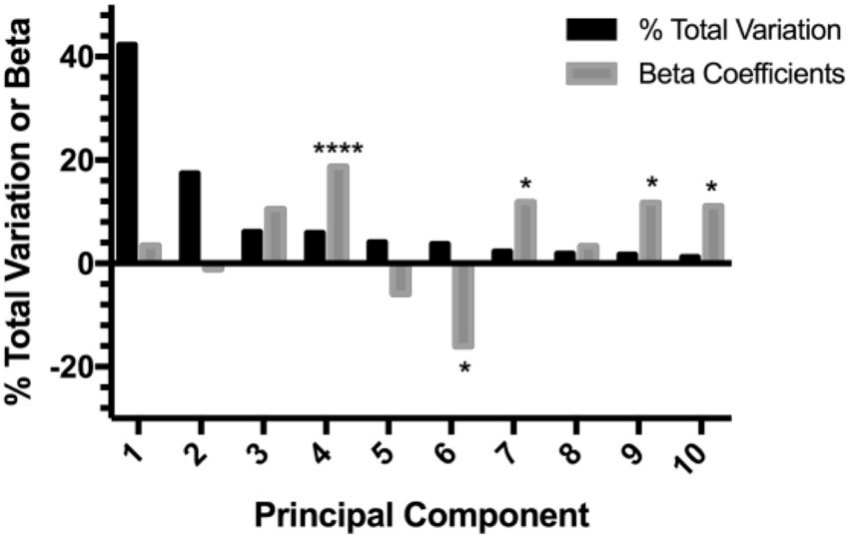
X-axis represent the principal component, and Y-axis represents the percent total variation of principal components or beta coefficient, respectively. Only the first 10 principal components (Pc) are displayed. Black bars represent the percentage of the sum of all possible eigenvalues; the Eigen value % of total variation. Gray bars represent the beta coefficients from regressions; model: (BMI_*i*_ = Age_*i*_ + Smoking_*i*_ + Pc_*k*_) where *i* = (*1*, *…*, *126*) and *k* = (*1*, *…*, *10*). *p-value < 0.05 and ****p-value < 0.0001. Body mass index (BMI).

Next, BMI was regressed on Pc scores derived from the 1,745 lipid abundances (Fig. 4). The first 10 Pc scores were analyzed individually and models were adjusted for age and smoking. The 4^th^ principal component accounted for 5.9% of Eigen value percent total variation and was positively associated (estimated effect = 18.7) with BMI (Bonferroni p < 0.05, data not shown). The 6^th^ principal component was inversely associated with BMI, and principal components 7, 8, 9, and 10 were positively associated with BMI; estimated effects: −16.1, 11.9, 11.8, and 11.0, respectively. In order to determine which lipids were driving the 4^th^ principal component scores, the 4^th^ principal component scores were regressed on the 1,745 lipids individually, and the top 10 beta coefficients positively and inversely associated with the 4^th^ principal component scores, and the lipids respective Bonferroni corrected p-values are presented in Supplementary Table 8.

### Structural identification of primary ids

The primary ids significantly associated with traits across several models were selected for structural identification. The primary ids, lipid common name, and list of models where each respective primary id was significant, are outlined in Table 3. PL head groups were determined as follows: PCs were confirmed by loss 60.02 Da (methyl formate), PEs were confirmed by presence of 140.01 *m/z* (ethanolamine phosphate), PIs were confirmed by presence of 241.01* m/z* (inositol phosphate-H_2_O), and PSs were confirmed by neutral loss of 87.03 Da (loss of serine). FA chains length and degree of saturation were determined as follows: C16:0 was confirmed by presence of 255.233 *m/z*, C17:0 was confirmed by presence of 269.248 *m/z*, C18:0 was confirmed by presence of 283.264 *m/z*, C18:1 was confirmed by presence of 281.248 *m/z*, C18:2 was confirmed by presence of 279.233 *m/z*, C18:3 was confirmed by presence of 277.217 *m/z*, C20:1 was confirmed by presence of 309.279 *m/z*, C20:2 was confirmed by presence of 307.264 *m/z*, C20:3 was confirmed by presence of 305.248 *m/z*, C20:4 was confirmed by presence of 303.233 *m/z*, C20:5 was confirmed by presence of 301.217 *m/z*, C22:5 was confirmed by presence of 329.248 *m/z*, and C22:6 was confirmed by presence of 327.233 *m/z*.

Eleven LPLs were associated with traits from several lipid classes including LPC, LPE, and LPS. These LPLs identified included: LPC(17:0), (18:1), (18:2), (18:3), (20:1), (20:2), (20:5), and (22:6); LPE(18:1) and (18:2); and LPS(20:4). The primary id X1.35_669.3989 fell within the retention time range of LPLs but did not exhibit a fragmentation pattern indicative of PLs (Supplemental Table 9) and was not available in lipid databases. The free FA palmitic acid was confirmed by 255.2331 *m/z* and matching the retention time (2.65 min) with a pure standard. The primary ids X4.18_794.5050, X5.43_1068.6601, and X10.11_1381.8480 did not have a fragment pattern indicative of PLs, although X5.43_1068.6601 did produce the m/z 1023.6949 indicative of formate (45.99 Da) loss.

Six PCs with ether linked chains were associated with several traits. These PCs with ether linked lipids included: PC(O-16:0_20:5), PC(O-16:1_18:2), PC(O-18:1_22:5), PC(O-16:0_18:2), PC(O-18:0_22:6), and PC(O-18:1_18:2). Ether linked chains in PCs were confirmed as follows: 466.3303 *m/z* for O-16:0, 464.3147 *m/z* for O-16:1, 494.3616 *m/z* for O-18:0, and 492.3460 *m/z* for O-18:1. In addition, there were several diacyl PLs associated with traits that contained FA chains ≥C18:0 and FA chains indicative of ω-6s, including: PC(18:2_18:2), PC(18:0_18:2), PC(20:1_20:4), PE(18:0_20:4), PC(18:0_22:5), PC(18:0_20:3), and PE(18:0_20:3). Since sphingolipids do not fragment well in negative mode, primary id X10.33_871.6904 was determined to be SM(d19:1/24:1) as follows: by confirmation of methyl formate loss of parent ion and presence of daughter ion 811.6074 *m/z* in negative mode, and the presence of 827.70 *m/z* (the SM(d19:1/24:1) [M + H]^+^) at 10.33 min in positive mode, and the fragments 278.28 *m/z* (indicative of SM d19:1 [long chain base-H_2_O]^+^) and 336.37 *m/z* (indicative of SM with 24:1 FA in positive mode). A complete table of the primary ids, common name, molecular adducts, theoretical *m/z*, and mass error (<0.05 for all identified lipids) are presented in Supplementary Table 10.

### Plasma PL FA chains are associated to FAs with specific double bond position and geometry in plasma PL

The untargeted analysis employed in our study does not determine the double bond geometry (i.e., cis/trans) or position (i.e., between carbons 6–7, 10–11, etc) on the unsaturated FA chains. The significant plasma PL structurally characterized in our current study (Table 3) were regressed on the % total plasma PL FA levels from our previous study^26^, since the plasma analyzed in our current and previous study were collected from the same patients at the same time point. Thus, researchers can infer the ω-family, and double bond geometry and position of the FA chains from our untargeted results. For instance, each one unit change in the % of LA (ω-6, C18:2^Δ9Z,12Z^) in plasma PL was associated with an average one unit change in LPC(18:2) and LPE(18:2) levels (Table 4). These 18:2 containing LPLs were not associated with the % of the trans-isomer linoelaidic acid (ω-6, C18:2^Δ9E,12E^) in plasma PL as outlined in Table 4. For each one unit change in the % of eicosapentaenoic acid (EPA, ω-3, C20:5^Δ5Z,8Z,11Z,14Z,17Z^) in plasma PL, the average change in mean LPC(20:5) and PC(O-16:0_20:5) levels were roughly one unit. Similarly, LPC(22:6), PI(16:0_22:6), and PC(O-18:0_22:6) were highly associated with the % of docosahexaenoic acid (DHA, ω-3,C20:5^Δ4Z,7Z,10Z,13Z,16Z,19Z^). PC(O-18:1_22:5) was highly associated with the % of docosapentaenoic acid ω-3 (DPA ω-3, C22:5^Δ7Z,10Z,13Z,16Z,19Z^) in plasma PL, and PC(O-18:1_22:5) and was not associated with % of DPA ω-6 (C22:5^Δ4Z,7Z,10Z,13Z,16Z^) in plasma PL (Table 4). On the other hand, PC(18:0_22:5) was highly associated with the % of DPA ω-6 (C22:5^Δ4Z,7Z,10Z,13Z,16Z^) in plasma PL and was not associated with % of DPA ω-3 (C22:5^Δ7Z,10Z,13Z,16Z,19Z^) in plasma PL. Each model in Table 4 was adjusted for BMI to account for the underlying association between BMI, and the plasma PL and % of FA isomer. The results presented in Table 4 were similar to the spearman correlations between each PL and the % of each FA isomer (Supplementary Table 11).

**Table 4:** Relationship between untargeted PL FA chains and the % of geometric and positional FA isomers in plasma PL.

Common name^a^	Palmitic^b^ C16:0	Stearic^b^ C18:0	Oleic^b^ C18:1	Elaidic^b^ C18:1	Eicosenoic^b^ C20:1	Linoleic^b^ C18:2	Linoelaidic^b^ C18:2	ALA^b^ C18:3	Eicosadienoic^b^ C20:2	DGLA^b^ C20:3	ARA^b^ C20:4	EPA^b^ C20:5	DPA ω-3^b^ C22:5	DPAω-6^b^ C22:5	DHA^b^ C22:6	Nervonic^b^ C24:1
LPC(20:5)	—	—	—	—	—	—	—	—	—	—	—	**1.02**	—	—	—	—
LPC(18:3)	—	—	—	—	—	—	—	**0.24**	—	—	—	—	—	—	—	—
LPC(22:6)	—	—	—	—	—	—	—	—	—	—	—	—	—	—	**0.96**	—
LPC(18:2)	—	—	—	—	—	**0.90**	0.01	—	—	—	—	—	—	—	—	—
LPE(18:2)	—	—	—	—	—	**1.33**	0.03	—	—	—	—	—	—	—	—	—
LPS(20:4)	—	—	—	—	—	—	—	—	—	—	**−0.85**	—	—	—	—	—
LPC(18:1)	—	—	**0.36**	0.02	—	—	—	—	—	—	—	—	—	—	—	—
LPE(18:1)	—	—	**0.48**	0.09	—	—	—	—	—	—	—	—	—	—	—	—
LPC(20:2)	—	—	—	—	—	—	—	—	0.28	—	—	—	—	—	—	—
LPC(17:0)	—	—	—	—	—	—	—	—	—	—	—	—	—	—	—	—
LPC(20:1)	—	—	—	—	0.11	—	—	—	—	—	—	—	—	—	—	—
Palmitic acid	1.24	—	—	—	—	—	—	—	—	—	—	—	—	—	—	—
PI(16:0_22:6)	0.09	—	—	—	—	—	—	—	—	—	—	—	—	—	**0.97**	—
PC(18:2_18:2)	—	—	—	—	—	**2.61**	**—0.29**	—	—	—	—	—	—	—	—	—
PC(O—16:0_20:5)	—	—	—	—	—	—	—	—	—	—	—	**0.99**	—	—	—	—
PC(18:1_18:2)	—	—	0.15	−0.17	—	**1.05**	**−0.14**	—	—	—	—	—	—	—	—	—
PC(O-16:1_18:2)	—	—	—	—	—	**0.93**	−0.12	—	—	—	—	—	—	—	—	—
PC(O-18:1_22:5)	—	—	—	—	—	—	—	—	—	—	—	—	**0.70**	0.21	—	—
PC(O-16:0_18:2)	—	—	—	—	—	**1.14**	−0.13	—	—	—	—	—	—	—	—	—
PC(18:0_18:2)	—	0.54	—	—	—	**1.04**	**−0.33**	—	—	—	—	—	—	—	—	—
PC(20:1_20:4)	—	—	—	—	0.31	—	—	—	—	—	−0.11	—	—	—	—	—
PE(16:0_18:1)	**2.53**	—	0.33	−0.21	—	—	—	—	—	—	—	—	—	—	—	—
PE(18:0_20:4)	—	**0.77**	—	—	—	—	—	—	—	—	0.12	—	—	—	—	—
PC(O-18:0_22:6)	—	—	—	—	—	—	—	—	—	—	—	—	—	—	**0.74**	—
PC(18:0_22:5)	—	**1.86**	—	—	—	—	—	—	—	—	—	—	−0.28	**0.63**	—	—
PC(18:0_20:3)	—	**0.76**	—	—	—	—	—	—	—	**0.68**	—	—	—	—	—	—
PC(O-18:1_18:2)	—	—	—	—	—	0.65	−0.11	—	—	—	—	—	—	—	—	—
PE(18:0_20:3)	—	**1.62**	—	—	—	—	—	—	—	**0.82**	—	—	—	—	—	—
SM(d19:1/24:1)	—	—	—	—	—	—	—	—	—	—	—	—	—	—	—	**0.81**

Each plasma PL was regressed on the % of each FA isomer in the plasma PL, and models were adjusted for BMI. This was performed to determine the relationship between the abundance of each plasma PL significantly associated with the responses in our study and the % of FA isomers in plasma PL from our previous study. For instance, LPC(18:2) and LPE(18:2) were significantly associated with the % of linoleic acid (LA, ω-6, C18:2^Δ9Z,12Z^) in plasma PL, whereas LPC(18:2) and LPE(18:2) were not correlated with the % of trans-isomer linoelaidic acid (ω-6, C18:2^Δ9E,12E^) in plasma PL. Numeric values represent the estimated effects (beta coefficients) and are bolded if p < 0.05. The plasma analyzed from both data sets were collected at the same time point from the same patients. Models were defined as: log(plasma PL) = log(% FA isomer in plasma PL) + BMI.

^a^The significant PL associated with the responses that were structurally characterized in our study. The experimental methodology employed was the UPLC-ESI-MS^E^ analysis of crude lipid extracts outlined in this manuscript.

^b^The geometric and positional FA isomers that were targeted in our previous study (Pickens *et al*. PLEFA. 2015). The experimental methodology employed was the FAME analysis of isolated plasma PL by GC-FID. The specific isomers investigated were: oleic acid (ω-9C18:1^Δ9Z^), elaidic acid (ω-9C18:1^Δ9E^), eicosaenoic acid (ω-9, C20:1^Δ11Z^), linoleic acid (LA, ω-6, C18:2^Δ9Z,12Z^), linoelaidic acid (ω-6, C18:2^Δ9E,12E^), dihomo-gamma-linolenic acid (DGLA, ω-6, C20:3^Δ8Z,11Z,14Z)^, arachidonic acid (ARA, ω-6, C20:4^Δ5Z,8Z,11Z,14Z^), docosapentaenoic acid ω-6 (DPA ω-6, C22:5^Δ4Z,7Z,10Z,13Z,16Z^), alpha-linolenic acid (ALA, ω-3, C18:3^Δ9Z,12Z,15Z)^, eicosapentaenoic acid (EPA, ω-3, C20:5^Δ5Z,8Z,11Z,14Z,17Z^), docosapentaenoic acid ω-3 (DPA ω-3, C22:5^Δ7Z,10Z,13Z,16Z,19Z^), docosahexaenoic acid (DHA, ω-3,C20:5^Δ4Z,7Z,10Z,13Z,16Z,19Z^), and nervonic acid (ω-9, C24:1^Δ15Z^).

## Discussion

This study characterized plasma PL profiles related to obesity and obesity-associated serum adipokines, cytokines, and the glycemic marker C-peptide. Specifically, we report that several classes of LPLs (i.e., LPC, LPE, and LPS) are inversely associated with BMI, WC, leptin, and C-peptide. Plasma ether linked PCs were also significantly decreased in obesity, including 16 and 18 carbon saturated and unsaturated ethers. On the other hand, total adiponectin, an anti-inflammatory adipokine inversely associated with obesity, was positively associated with LPLs and C18:2 containing PLs. Overall we report that C18:2 is lower in several lipid classes (i.e., LPLs, diacyl PCs, and ether linked PCs) in obese individuals. The significant PL containing C18:2 chains were highly associated with the % of LA (ω-6, C18:2^Δ9Z,12Z^) in plasma PL. We previously reported in this study population, that median plasma PL LA (ω-6, C18:2^Δ9Z,12Z^) levels are roughly 3% lower in our obese patients compared to lean^26^. Results of our current study indicate that plasma PL FA differences occur in specific PL species, and since obesity is associated with altered lipid metabolism, our results provide insight into metabolic pathways that may be of interest for future research.

Associations between LPLs and overweight/obesity are well described, in particular the decrease in LPC(18:2)^15, 16^. After weight loss in obese individuals there is an increase in LPCs, in particular C18:2, suggesting the inverse association between LPLs and obesity could be related to diet or weight loss^29, 30^. Interestingly, the seminal paper by Pietilainen *et al*. reported in monozygotic twins that obesity was associated with increases serum LPCs, including LPC(18:2), and decreases in serum ether lipids, which suggests that changes in LPL profiles are likely associated with genetic factors related with obesity rather than environmental and lifestyle factors^31^. In experimental models with high fat diets (HFD), there are rapid decreases in plasma LPC(18:1), (18:2), (20:1), and (20:5)^25^. Del Bas *et al*. recently reported, in a randomized control trial, that ω-3 PUFA supplementation only increases ω-3s in LPLs and lowers ω-6s in LPLs, but has no effect on overall LPL concentrations in overweight individuals^18^. Therefore, the observed decrease in plasma LPLs may suggest that our obese patients were consuming high-fat diets prior to study recruitment, and we did not assess dietary status in our study. C-peptide, a marker of insulin production, was also inversely associated with LPLs including LPC(18:2). Higher C-peptide indicates a higher production of insulin^32^. Our patient exclusion criteria excluded individuals with type-1 or type-2 diabetes, and those being treated for insulin resistance. Lower levels of plasma LPCs are associated with obese individuals^25^, obese individuals with type-2 diabetes^25^, and have been associated with insulin resistance independent of obesity^33^. Interestingly, LPCs activate PPARδ to reduce skeletal muscle inflammation and ER stress^34^, and accumulations of fat in skeletal muscle^35^ and liver^36^ are associated with decreased insulin sensitivity.

Studies have shown that fat accumulation in the liver occurs independent of obesity and is more associated with insulin resistance^37^. Insulin resistance is associated with increased hepatic fat accumulation, which alters hepatic lipoprotein secretion such as increasing secretion of low-density and very low-density lipoproteins, and decreasing secretion of high-density lipoproteins [reviewed in detail ref. 38]. In fact, high-density lipoprotein particles are rich in LPLs and obesity is associated with decreases in high-density lipoproteins^39^. We did not assess lipoprotein levels or screen our patients for fatty liver disorders, however, LPC(18:2) and ether lipid concentrations are inversely associated with liver fat percentages and are predictive of NAFLD^40^. Furthermore, the enzyme LCAT is largely responsible for generating plasma LPLs, and LCAT is present in low-density lipoproteins, which are increased in individuals with obesity, insulin resistance, and fatty liver disorders [reviewed in detail refs 8 and 38]. Sansone *et al*. reported that intra-patient concentrations of LA (C18:2^Δ9Z,12Z^) were higher in plasma cholesterol esters and lower in RBC PLs, suggesting that LA is being shunted from the PL to neutral lipids such as cholesterol esters^41^. It is likely our observed decrease is plasma LPLs could be associated with a combination of factors including dietary intake, altered lipoprotein metabolism, shunting of specific FAs from PL to cholesterol esters, insulin resistance, and fatty liver.

Overall most of the significant plasma PL FAs differences in our results were PUFAs, in particular C18:2 which decreased in LPLs, ether linked PCs, and diacyl PCs. The specific double bond location and geometry of unsaturated FA chains are associated with metabolic diseases^42^. Correlations between the significant PL containing 18:2 chains and FA data from our plasma PL FAME analysis indicated that LA (C18:2^Δ9Z,12Z^) is likely decreasing in these specific LPLs, ether lipids, and diacyl PCs (Table 4). A recent meta-analysis reported that long chain PUFA profiles, in particular ω-6s, are dysregulated in overweight and obese individuals^6^. In obesity, decreases in LA are associated with increases in LA-derived DGLA by the enzyme delta-6-desaturase (D6D). Taking into account the results from Sansone *et al*.^41^, LA decreases in obesity are likely due to a combination of LA shunting to neutral lipid species and increased metabolism of LA to long chain ω-6 PUFAs through D6D. We have previously reported in our study population that PL DGLA levels and D6D enzyme activity estimates (EAE), D6D EAE = ratio of DGLA/LA, are increased in obese individuals^26^. In our current study we report C20:3 (i.e., DGLA) is increased specifically in PC(18:0_20:3) and PE(18:0_20:3), and these PL contains 20:3 chains are highly associated with the % of DGLA (ω-6, C20:3^Δ8Z,11Z,14Z^) in plasma PL. In addition, we have also reported in our study population that C-peptide concentrations are inversely associated with plasma PL delta-5-desaturase (D5D) EAEs (i.e., the ratio of ARA/DGLA) even after adjusting for obesity (i.e., BMI and WC)^27^. In our current study, for each one unit change in PE(18:0_20:3), the average change in mean C-peptide concentrations was roughly 2.13 units (Supplementary Table 6). It is likely that PLs such as PE(18:0_20:3) and PC(18:0_20:3) are important lipids contributing to the increases in PL DGLA levels that is observed in numerous obesity related studies (reviewed in detail refs 2 and 6).

The purpose of our study was to identify specific plasma PL species with FA differences associated with traits (i.e., BMI, WC, serum C-peptide), and assess the contribution of the entire plasma lipidome on the variation of these traits, in a cross-sectional study (n = 126, >96% Caucasian males, ages 48–65). We recognize the generalizability of our study and these results should be verified in larger, more diverse populations. We acknowledge not assessing diet, % energy from fat, and supplement usage are limitations to our study. Although our patient exclusion criteria excluded those with diabetes and those being treated for insulin resistance, it is possible some of our obese patients were insulin resistant. Our untargeted analysis identified FA chains esterified in plasma PL, and the double bond position and geometry could not be determined using our method. Therefore, we correlated patient’s PL FAME levels with their levels of significant untargeted lipids, so the double bond geometry and position can be inferred. In conclusion, the whole plasma lipidomic profiles accounted for over 60% of the inter-individual variation in obesity. Our results are in agreement that obesity is associated with decreases in LPLs^15–18, 25^, ether linked lipids^31, 43–45^, and an overall decrease in plasma PL LA^2, 6, 41^. We report plasma PL LA likely decreases in the following lipid species: the LPLs LPC(18:2) and LPE(18:2), the diacyl PCs PC(18:0_18:2), PC(18:1_18:2), and PC(18:2_18:2), and the ether linked PCs PC(O-16:0_18:2), PC(O-16:1_18:2), and PC(O-18:1_18:2). Future research should quantify the levels of these specific lipids using synthetic standards and determine their feasibility for use in clinical diagnostics screenings for obesity-associated co-morbidities.

## Materials and Methods

### Study population

Data are described elsewhere^26, 46–50^. In brief, healthy and asymptomatic male subjects (n = 126, >96% Caucasian) 48 to 65 years of age were enrolled between 2009 and 2011 in a cross-sectional study. Data are comprised of clinical metadata on individuals’ co-morbidities, family history, and use of medications. Individuals with the following medical conditions were excluded: (1) cancer in the previous two years, (2) surgery in the previous two years, (3) type-1 and type-2 diabetes, and (4) autoimmune diseases. Immediately after enrollment, trained staff collected anthropometric measurements and venous blood of study participants. In brief, body mass index (BMI) was assessed by recording patient’s height using a stadiometer and weight using digital platform scale. The plasma fraction was separated from whole blood by centrifugation and stored at −80 °C until time of analysis. Smoking status was assessed as “ever smoked” or “never smoked”. A previous complete description of this study can be found elsewhere^46^. All participants provided written informed consent, and all experiments were performed in accordance with relevant guidelines and regulations. The study was approved by the Biomedical and Health Institutional Review Board of Michigan State University (IRB# 08-786).

### Plasma preparation

A detailed list of chemicals, solvents, and internal standards used in plasma lipid extractions are presented in Supplementary Table 1. Plasma lipid extraction was performed following a Waters application note^51^, but modified as specified. A quality control (QC) sample was made by pooling 10 μL plasma from all 126 samples, and this QC sample was extracted and processed alongside individual samples. Plasma samples were thawed on ice. A 50 μL aliquot of each plasma was transferred to a labeled microcentrifuge tube containing 200 μL of the extraction solution (2:1 v/v, 100 μg/μL BHT, 5 ng/μL PLs and 0.2 ng/μL Cer). These mixtures were then incubated on ice for 15 minutes, then vortexed for 30 s. After vortexing, protein crash was performed by centrifugation in an accuSpin™ Micro R centrifuge at 13,000 × g for 5 min at 4 °C (Fisher Scientific, Waltham, MA). Following centrifugation, each lower organic phase was collected and transferred to new labeled microcentrifuge tube, evaporated to dryness in a Savant SpeedVac (ThermoQuest, Holbrook, NY) for 3 h, with no applied heating, then stored under high-purity nitrogen at 4 °C for no longer than 1 day. Dried residues were re-dissolved in 500 μL HPLC-grade isopropanol:acetonitrile:water (2:1:1 v/v/v), then vortexed gently. Re-dissolved extracts were again centrifuged 13,000 × g for 5 min at 4 °C (Fisher Scientific). After centrifugation, 100 μL drawn from the top of the supernatant was transferred to an amber autosampler vial with glass insert, and the vials were purged with high-purity nitrogen and sealed.

### Lipidomic profiling by UPLC-MS/MS analyses

Mass spectrometric analysis was accomplished following Waters application note^51^, but modified as specified. In brief, reverse phase ultra-performance liquid chromatography (UPLC)-ESIneg-MS/MS was performed on a Waters ACQUITY UPLC (Waters, Milford, MA) coupled to a Waters Xevo G2-XS quadrupole time-of-flight mass spectrometer (Waters). Chromatographic separation was performed with an ACQUITY UPLC CSH C18 1.7 µm 2.1 × 100 mm column (Waters) held at 55 °C. The autosampler (Waters) temperature was held at 10 °C. The UPLC method was shortened to 15 min and the mobile phases and gradients, along with mass spectrometer settings are outlined in Supplementary Table 1. Data acquisition was performed using MS^E^ in continuum mode with leucine enkephalin as lock mass for mass correction. The QC sample was injected after every 10th sample throughout the entire analysis.

### Data alignment and processing of plasma lipids

Results from UPLC-MS^E^ analyses were imported into Progenesis QI v2.0 (Nonlinear Dynamics, Durham, NC). Peaks were aligned using one of the QC samples as references. Peak picking was performed under normal conditions, collecting signals from 4802 ions. The Progenesis QI generated data set was subjected to relative mass defect filtering to narrow ions to those of potential lipids^52^, and ions with relative mass defects <350 and >950 parts-per-million (ppm) were excluded from the data set. The mass defect filtered data set was then imported into R for multivariate statistical analyses. Since large -omic data sets often have 10–20% missing values^53, 54^, 1126 ions with >20% missing values were excluded from the data set, the 10 IS were split from the data set, and peak areas for the remaining 1,745 ions were mean imputed. Next, due to a time of injection affect (details are presented in Supplementary Table 1), the data matrix peak areas were normalized to the IS PC(8:0/8:0) signal. After IS normalization of data matrix, the coefficient of variation between QC injections was <5% for the most significant lipids outlined in this manuscript.

### Plasma PL FAME data

Plasma was extracted and PL were isolated as previously described^55, 56^. Experimental methods and PL FAME acquisition are outlined in detail as previously described^26^.

### Statistical analyses

Our study was powered (0.8) to detect differences in plasma FAs, as previously described^49^. Statistical analysis of the data was performed using R v3.2.2^57^, and all R-code used in these analyses are included in the Supplementary Table 12 and publicly available at https://github.com/AustinPickens/Untargeted-Lipidomics. The responses used in the statistical analyses were the traits: BMI, waist circumference (WC), log transformed serum leptin, log transformed serum total adiponectin, log transformed serum c-peptide, log transformed serum interleukin-6 (IL-6), log transformed serum tumor necrosis factor-alpha (TNF- α), log transformed serum interferon gamma-induced protein-10 (IP-10), and serum monocyte chemoattractant protein-1 (MCP-1). The analysis consisted of two parts: (1) Single lipid regressions on each trait associating the abundance of the lipid in plasma with the responses, to identify individual plasma lipids significantly associated with each response; (2) High dimensional analyses including principal components (Pc), singular value decomposition (SVD), and a model regressing the responses in all lipids in plasma, to identify whether the inter-individual differences can be attributed to the lipidomic profile. P-value correction was performed according to Benjamini-Hochberg false discovery rate (BH FDR) and Bonferroni^58, 59^.

#### Single Lipid Regressions

Response variables include: BMI, WC, log transformed leptin, log transformed total adiponectin, log transformed c-peptide, log transformed IL-6, log transformed TNF- α, log transformed IP-10, and MCP-1. We herein refer to these responses as traits, and these traits consist of phenotype *y*_*i*_(*i* = 1, …, 126) indexed by individuals (*i*) and the set of predictors coming from the lipidomic data as well as clinical covariates including age of the participant (*a*_*i*_) 56.9 ± 4.7 (mean ± s.d.), the status of smoking behavior (*s*_*i*_) 25.40% ever smoked, 57.14% never smoked, 17.46% smoking unknown. In each regression only one of the lipids was included from the pareto standardized lipidomic data (*l*_*ij*_). The statistical model was adjusted for each lipid, *j* = 1, …, 1745 as follows in Eq. (1):

1 $${y}_{i}={\beta }_{0j}+{a}_{i}{\beta }_{1j}+{s}_{i}{\beta }_{2j}+{l}_{ij}{\beta }_{3j}+{\varepsilon }_{ij},$$

let *β*_0*j*_ be a general intercept, and *β*_1*j*_, *β*_2*j*_ and *β*_3*j*_ the corresponding regression coefficients and *ε*_*ij*_ a random residual following a IID normal distribution centered at zero. A total of 1,745 regressions, one per lipid, were adjusted in Eq. (1).

#### High dimensional analyses

The lipidomic data is a high-dimension dataset with a higher number of parameters to estimate (p = 1745) then observations (n = 126); for example p ≫ n. Thus, the following analysis were performed: A) Pc derived from lipidomic data abundances, B) SVD derived from the lipids estimated effects obtained in the single marker regressions, and C) whole lipidome regressions, as described in refs 60–62.

A) The transposed cross product (i.e., X′X) of the *nxm* (*n* = 1, …, 126; *m* = 1, …, 1745) matrix of lipidomic abundances was used to produce an *nxn* matrix of distances. Next, an Eigen value decomposition of the *nxn* matrix was employed to generate Eigen values and Pc scores for each of the Pcs-derived from lipid abundances. There were 126 Pcs generated and we evaluated only the first ten Pcs. The effects of each Pc were evaluated by regressing BMI against Pc scores individually. The statistical model was adjusted for each Pc, *k* = (1, …, 10) as follows in Eq. (2):
  2 $$BM{I}_{i}\,={\beta }_{0k}+{a}_{i}{\beta }_{1k}+{s}_{i}{\beta }_{2k}+\,P{c}_{ik}{\beta }_{3k}+{\varepsilon }_{ik}$$
  let BMI be the phenotype (*i* = 1, …, 126) indexed by individuals (*i*) and the set of predictors coming from the Pc scores derived from lipid abundances and let *β*_0*j*_ be a general intercept, and *β*_1*k*_, *β*_2*k*_, and *β*_3*k*_ the corresponding regression coefficients and *ε*_*ik*_ a random residual following a IID normal distribution centered at zero. A total of 10 regressions, one per Pc, were adjusted in Eq. (2). Finally, since Pc4 was highly associated with BMI, Pc4 scores were regressed on the individual lipids, one at a time to identify the lipids driving Pc4. The statistical model adjusted for each lipid, *j* as follows in Eq. (3):
  3 $$Pc{4}_{i}\,={\beta }_{0j}+\,{l}_{ij}{\beta }_{1j}+{\varepsilon }_{ij}$$
  in this analysis, Pc4 loadings are derived from lipid abundances and the set of predictors coming from lipidomic data and let *β*_0*j*_ be a general intercept and *β*_1*j*_ the corresponding regression coefficients and *ε*_*ij*_ a random residual following a IID normal distribution centered at zero. A total of 1,745 regressions, one per lipid, were adjusted in Eq. (3).
B) Data were multidimensional, thus, a biplot was used to extend the bivariate scatter plot. Biplots allow displaying information on samples and variables simultaneously. Here, a matrix with the lipid effects was constructed with lipids in the rows (beta coefficients of the 1,745 lipids) and 9 different traits in the columns (i.e., BMI, WC, leptin, total adiponectin, C-peptide, IL-6, IP-10, and MCP-1). That matrix was decomposed in three using SVD, and the biplot results, displayed in Fig. 3, are the first two vectors that spanned the rows based on the column space of the SVD^63^.
C) Finally, the lipidome was fully accounted for in a whole genome regression analysis^61, 62^. The probabilistic model assumed used in each of the responses was as follows in Eq. (4),

4 $${y}_{i}={\beta }_{0}+a{\beta }_{1}+s{\beta }_{2}+\sum _{j=1}^{j=1745}\,{x}_{ij}{\alpha }_{j}+{\varepsilon }_{i},$$

where ${u}_{i}={\sum }_{j=1}^{j=1745}{x}_{ij}{\alpha }_{j}$ represents the total effect of the lipids on trait *y*_*i*_, *x*_*ij*_ is the lipidomic abundance for participant *i* and lipid *j*, and α_j_ is the estimated effect. In Eq. (4), assume that the responses consist of the traits indexed by individuals (*i*) and the set of predictors coming from the lipidomic data. *u*_*i*_ follows a normal distribution centered zero with variance covariance ${u}_{i} \sim N(0,G{\sigma }_{g}^{2})$, and *G* is an *nxn* matrix of distances to measure similarities between participants with respect to their lipid profiles, as previously described^64^. These analyses were conducted using the R-package BGLR^65^, using a long Markov Chain of 200,000 iterations and 50,000 samples were discarded for burn in. Inference was done based on one of every 5 samples of the last 150,000. High dimensional regressions were adjusted to the lipidomic data as describe before for other omics^60, 66^.

### Structural Identification of Plasma Lipids

Lipids in this study are presented in the form of Xretention time_mass-to-charge ratio (i.e., X1.17_564.3289 [1.17 mins, 564.3298 *m/z*]). The structural of identification of plasma lipids was performed if the integrated peak areas for the ion: were significant in two or more models from single marker regression analyses of responses, or had radii ≥0.15 in SVD analysis (Fig. 3, and Supplementary Table 7), or was one of the top ten lipids positively and inversely associated with Pc4 (Supplementary Table 8), and the *m/z* exhibited a fragmentation pattern indicative of a free FA, LPL, or PL species. Since the objective of this study was to identify free FA and PL differences related to obesity and obesity-associated adipokines, significant lipid ions that did not have fragment patterns matching free FA and PLs are listed as unknown (Table 3), and their spectra are listed in Supplementary Table 9. Plasma lipids meeting our criteria for identification that are not listed in Table 3 were too low in abundance to obtain a useful MS/MS spectrum and are listed as too low to identify (TLTI). Lipid structure identification was confirmed using Lipid Maps (http://lipidmaps.org) and Human Metabolome database (http://www.hmdb.ca), and manual evaluation of MS/MS spectra. The “_” denotation is used between FAs in glyceroPL structures, since the FA position (i.e., either sn1 and sn2) were not determined.

## Electronic supplementary material

Supplementary Information
