# Supplementary material for: Obesity, adipokines, and C-peptide are associated with distinct plasma phospholipid profiles in adult males, an untargeted lipidomic approach
Source: Sci Rep. 2017 Jul 24;7:6335. doi: 10.1038/s41598-017-05785-0 (PMC5524758; doi:10.1038/s41598-017-05785-0)
Supplement: Supplementary file 1 — Supplementary Information [file 41598_2017_5785_MOESM1_ESM.pdf]

Supplementary Information

Title: Obesity, adipokines, and C-peptide are associated with distinct plasma phospholipid profiles in adult males, an untargeted lipidomic approach.

C. Austin Pickens[1], Ana I. Vazquez[2], A. Daniel Jones[3,4], Jenifer I. Fenton[1]

Supplementary Table 1: Chemicals and solvents, chromatographic and mass spectrometric analysis parameters, and identification and correction of time of injection effect.

Chemicals and solvents

High performance liquid chromatography-grade (HPLC) solvents were used for the extraction and resuspension of all samples analyzed. The following solvents were used in the analysis: HPLC-grade methanol (Lot#: 0000118827, J.T. Baker, Phillipsburg, NJ), HPLC-grade chloroform (Lot#: 55296, Omnisolv, Charlotte, NC), HPLC-grade water (Lot#: 0000063121, J.T. Baker), HPLC-grade isopropanol (Lot#: SHBC6903V, Sigma-Aldrich, St. Louis, MO), and HPLC-grade acetonitrile (Lot #: 53324, EMD chemicals, Gibbstown, NJ).

Internal standards for each lipid class were selected based on commercial availability at time of analysis. The internal standards used in this study are as follows: PE(16:0-d_{31}/18:1) [Product#:110921; Lot#: LM160D31-181PE-33], PG-d_5(16:0/18:1) [Product#: 110919; Lot#:LM160-181PGD5-10], PS(16:0-d_{31}/18:1) [Product#: 110922; Lot#: LM160D31-181PS-25], LysoPE(13:0) [Product#: 110696; Lot#: LM130LPE-10], PC(24:0/24:0) [Product#: 110929; Lot#: LM240PC-28], Cer(d18:1/25:0) [Product#: LM-2225; Lot#: LM5-139D] (Avanti Polar Lipids, Alabaster, AL), and PC(8:0/8:0) [Product#: 10009874; Lot#181547-14] (Cayman Chemical, Ann Arbor, MI). The antioxidant butylated hydroxytoluene (BHT) (Sigma-Aldrich) was present in all solvents at a concentration of 0.1%.

Internal standards and extraction solution

A mixture of internal standard (IS) containing PE(16:0-d_{31}/18:1), PG-d_5(16:0/18:1), PS(16:0-d_{31}/18:1), LysoPE(13:0), PC(24:0/24:0), Cer(d18:1/25:0) (Avanti Polar Lipids), and PC(8:0/8:0) (Cayman Chemical) was prepared. This IS mixture was added to an extraction solution composed of HPLC-grade chloroform:methanol (2:1 v/v, Omnisolv, J.T. Baker) with 0.1% BHT (100 µg/µL, Sigma-Aldrich). The final concentration of IS in the extraction solution was 5 ng/µL for all PLs and 0.2 ng/µL for Cer.

UPLC MS/MS Analysis and Parameters

The instrument used in plasma lipid separation was a Waters ACQUITY UPLC using ACQUITY UPLC CSH C18 1.7 µm 2.1x100mm column (Waters, Milford, MA). The column was washed prior to analysis for 1.5 h with 50% dichloromethane and 50% 2-propanol, followed by 1.5 h with 100% 2-propanol. The UPLC method used in this analysis performed was based on the Waters application note: Issac et al. Lipid Separation using UPLC with Charged Surface Hybrid Technology. The UPLC method was modified to shorten the run time to 15 min.

UPLC Parameters

Parameter	Setting
Run Time:	15.00 min
Solvent A:	Acetonitrile/water (60:40) + 10 mM ammonium formate
Solvent B:	Isopropanol/acetonitrile (90:10) + 10mM ammonium formate
Column Temperature:	55.0 °C
Injection Volume (μL):	10.00
Equilibration Time:	0.1 min

UPLC Gradient Table

Time (min)	Flow Rate	%A	%B	Curve
Initial	0.400	60	40	Initial
1.50	0.400	57	43	6
1.60	0.400	50	50	1
9.00	0.400	57	43	6
9.08	0.400	30	70	1
13.50	0.400	1	99	6
13.60	0.400	60	40	6
15.00	0.400	60	40	1

The MS/MS analysis of plasma lipids was performed using a Waters Xevo G2-XS quadrupole time-of-flight mass spectrometer (Waters). Data acquisition was performed using MS^E in continuum mode with lock mass application. Source parameters are as follows:

Mass Spectrometer Source and Optics Settings

Parameter	Setting
Analyzer	Sensitivity mode
Capillary (kV)	1
Sampling Cone (kV)	10
Source Temperature (°C)	110
Source Offset	80
Desolvation Temperature (°C)	350
Cone Gas Flow (L/Hr)	25
LM Resolution	4.7
HM Resolution	15

Three MS functions were used to obtain MS/MS spectra and correct for mass drift: Function1 was used to obtain a parent ion spectra of lipids; Function 2 was used to obtain a fragmentation spectra of parent lipid ions; and Function 3 was used to measure leucine enkephalin as lock mass for mass correction.

Mass Analyzer Settings and Collison Energies

**Function 1: TOF Parent Ion Function**	
Survey Start Time (min)	0.0
Survey End Time (min)	15.0

Survey Ion Mode	ES Mode
Survey Polarity	Negative
[PARENT MS SURVEY]	
Survey Start Mass	60
Survey End Mass	1800
Parent Survey High CE (V)	30.0
Parent Survey Low CE (V)	10.0
**Function 2: TOF Fragmentation Function**	
Survey Start Time (min)	0.0
Survey End Time (min)	15.0
Survey Ion Mode	ES Mode
Survey Polarity	Negative
[PARENT MS SURVEY]	
Survey Start Mass	60
Survey End Mass	1800
Parent Survey High CE (V)	90
Parent Survey Low CE (V)	20
**Function 3: TOF Lockmass Function**	
Survey Start Time (min)	0.0
Survey End Time (min)	15.0
Survey Ion Mode	ES Mode
Survey Polarity	Negative
[PARENT MS SURVEY]	
Survey Start Mass	60.0
Survey End Mass	1800.0
Parent Survey High CE (V)	30
Parent Survey Low CE (V)	10

Progenesis QI Data Importation

Raw UPLC-MS/MS data files were imported into Progenesis QI for data alignment and processing. The following adducts were selected based on the sample matrix (i.e., chloride), mobile phases (i.e., formate), and contaminants (i.e., trifluoroactetate and nitrate) that may have been present in either the samples or the mass analyzer at time of analysis. The following adducts were included for deconvolution of M, 2M, and 3M *m/zs*: M-H, M+formate-H, M-methylformate-H, M+Cl-H, M+trifluoroacetate-H, M-methyltrifluoroacetate-H, and M+nitrate-H. After peaking picking and deconvolution by Progenesis QI, a total of 4802 m/zs were identified. The data was first exported to EZ-Info v3.0 (Umetrics, San Jose, CA), then to Excel (Microsoft, Redmond, WA) for relative mass defect filtering.

Time of Injection Effect

After initial data processing of mass defect filtered dataset, we investigated batch and time of injection effects in the data. The variable time of injection was created by multiplying the samples file number by 15 mins, thus, yielding the samples time of injection relative to the to the first injection of the analysis. We observed a time of injection effect after plotting principal components (Pc) 1 and 2. There were distinct clusters of samples based on their time of injection (mins) during the mass spectrometric analysis. In addition, the Pc 1 and 2 score were significantly associated with time of injection in regression analyses.

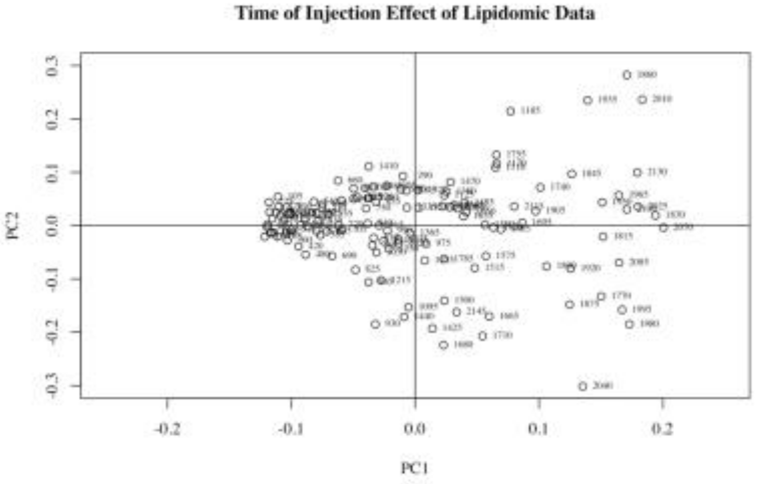

Principal components derived from patient (n=126) lipidomic profiles. Number listed next to samples indicate the samples time of injection in mins relative to the first injection of analysis. Samples injected earlier in the analysis clustered tightly together, while samples injected later in the analysis drifted.

Next, to further examine the time of injection effect, we plotted the range of intensities for each plasma lipid in the 126 samples.

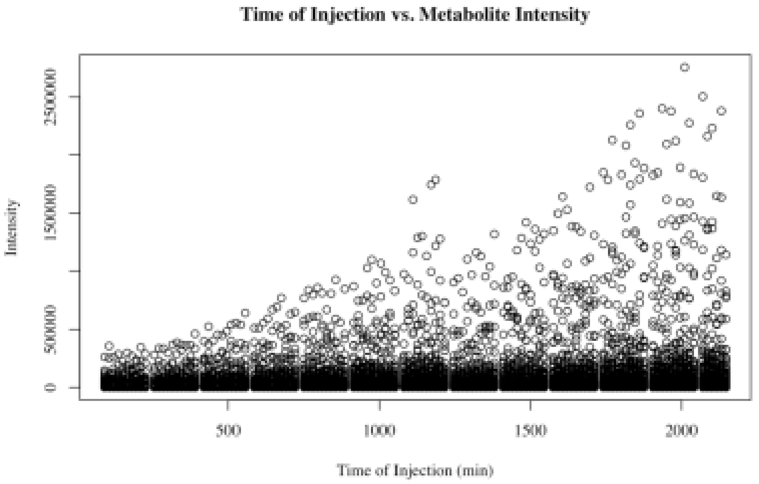

Figure displays time of injection and the range of plasma lipid intensities for each sample (n=126) analyzed. As time of injection increased, so did the range of metabolite intensities,

indicating ion suppression throughout analysis. We determined the time of injection effect was due to ion suppression by trifluoroacetate contamination in the mass analyzer. Since a majority of plasma phospholipids are PCs, we normalized our entire data matrix by the IS PC(8:0/8:0). IS normalization of the data matrix removed the time of injection effect on plasma lipids.

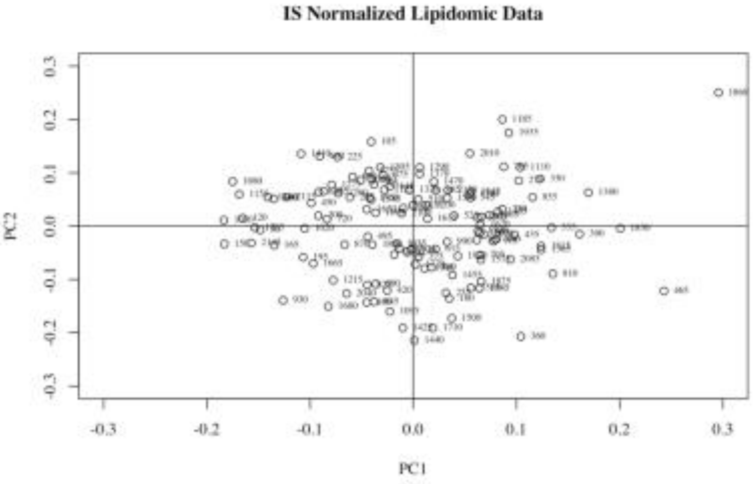

Principal components derived from patient (n=126) IS normalized lipidomic profiles. Number listed next to samples indicate the samples time of injection in minutes. Normalization of the data matrix with IS PC(8:0/8:0) removed the time of injection effect. Next, to further examine if normalizing the data matrix removed the time of injection effect, we plotted the range of intensities for each plasma lipid in the 126 samples

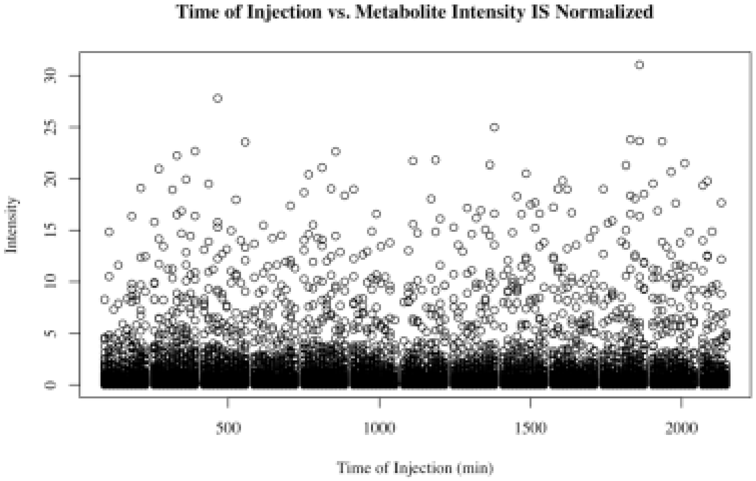

Above figure displays time of injection and the range of plasma lipid intensities for each sample (n=126) analyzed. After IS normalization of the data matrix, the time of injection effect was removed.

Supplementary Table 2: Table of lipids significantly associated with BMI with FDR p-values <0.05. Beta coefficients, p-values, Benjamini-Hochberg FDR p-values, and Bonferroni p-values listed.

Primary ID	Beta	p-value	FDR p-value	Bonferroni p-value
X1.17_564.3289	-5.25	1.48E-10	2.59E-07	2.59E-07
X1.42_566.3497	-10.78	1.30E-06	1.14E-03	2.28E-03
X1.21_476.2768	-14.67	4.03E-06	2.35E-03	7.04E-03
X1.51_592.3513	-115.61	1.06E-05	3.92E-03	1.86E-02
X1.03_562.3132	-44.59	1.12E-05	3.92E-03	1.96E-02
X5.23_826.5592	-7.70	1.47E-05	4.28E-03	2.57E-02
X7.15_786.5626	-6.28	1.95E-05	4.87E-03	3.41E-02
X1.59_554.3446	-23.44	4.62E-05	1.01E-02	
X4.18_794.5050	-32.49	5.40E-05	1.05E-02	
X1.49_478.2927	-17.91	8.28E-05	1.44E-02	
X2.63_393.2768	14.21	2.19E-04	3.39E-02	
X9.42_814.5927	-11.67	2.33E-04	3.39E-02	
X1.08_612.3280	-13.31	2.56E-04	3.44E-02	
X8.93_856.6036	3.98	2.76E-04	3.45E-02	
X6.50_828.5729	-1.80	3.35E-04	3.90E-02	
X3.52_421.3076	85.75	4.46E-04	4.34E-02	
X1.22_544.2648	-36.54	4.50E-04	4.34E-02	
X2.89_493.3353	-33.94	4.72E-04	4.34E-02	
X5.27_1031.496	-56.39	4.80E-04	4.34E-02	
X4.36_825.5432	-29.25	5.16E-04	4.34E-02	
X2.72_639.5552	11.27	5.42E-04	4.34E-02	
X5.43_1068.6601	-28.22	5.51E-04	4.34E-02	
X8.90_880.6027	7.18	5.96E-04	4.34E-02	
X5.24_1108.4552	-21.48	5.97E-04	4.34E-02	
X5.24_952.4993	-16.66	6.86E-04	4.78E-02	

Supplementary Table 3: Table of lipids significantly associated with waist circumference with FDR p-values <0.05. Beta coefficients, p-values, Benjamini-Hochberg FDR p-values, and Bonferroni p-values listed.

Primary ID	Beta	p-value	FDR p-value	Bonferroni p-value
X1.17_564.3289	-5.36	2.02E-08	3.52E-05	3.52E-05
X7.15_786.5626	-8.14	9.95E-07	8.68E-04	1.74E-03
X1.42_566.3497	-11.92	3.06E-06	1.78E-03	5.35E-03
X9.42_814.5927	-15.22	2.29E-05	8.85E-03	3.99E-02
X1.51_592.3513	-125.77	2.99E-05	8.85E-03	
X8.93_856.6036	5.17	3.04E-05	8.85E-03	
X1.91_594.3757	-67.46	4.25E-05	1.06E-02	
X11.11_1012.7635	-44.18	7.56E-05	1.58E-02	
X5.90_810.5600	-17.97	8.15E-05	1.58E-02	
X1.03_562.3132	-45.07	1.19E-04	1.81E-02	
X8.85_788.5789	-13.63	1.24E-04	1.81E-02	
X8.61_766.5363	4.13	1.31E-04	1.81E-02	
X5.43_1068.6601	-35.46	1.35E-04	1.81E-02	
X5.23_826.5592	-7.76	1.53E-04	1.91E-02	
X10.33_871.6904	-44.98	1.67E-04	1.92E-02	
X6.50_828.5729	-2.15	1.82E-04	1.92E-02	
X7.60_814.5916	-16.59	1.87E-04	1.92E-02	
X10.16_816.6098	-32.01	2.03E-04	1.97E-02	
X1.59_554.3446	-24.37	2.32E-04	2.12E-02	
X7.32_864.6056	-24.59	2.72E-04	2.12E-02	
X0.99_586.3136	-24.35	2.77E-04	2.12E-02	
X4.18_794.5050	-33.65	2.78E-04	2.12E-02	
X9.01_1128.5388	41.17	2.79E-04	2.12E-02	
X8.90_880.6027	8.58	3.18E-04	2.31E-02	
X3.52_421.3076	98.52	4.15E-04	2.90E-02	
X9.14_790.5935	-10.65	4.36E-04	2.93E-02	
X10.95_650.6069	24.14	4.62E-04	2.99E-02	
X10.11_1381.848	-42.51	4.80E-04	2.99E-02	
X1.08_612.3280	-14.34	5.93E-04	3.55E-02	
X6.26_834.5625	-16.98	6.11E-04	3.55E-02	
X10.16_826.6359	-30.64	7.08E-04	3.98E-02	
X7.02_772.5264	-10.17	7.36E-04	4.02E-02	
X1.21_476.2768	-12.46	7.90E-04	4.18E-02	
X7.47_788.5783	-9.13	9.57E-04	4.91E-02	
X8.61_1038.473	32.37	9.91E-04	4.94E-02	

Supplementary Table 4: Table of lipids significantly associated with serum leptin with FDR p-values <0.05. Beta coefficients, p-values, Benjamini-Hochberg FDR p-values, and Bonferroni p-values listed.

Primary ID	Beta	p-value	FDR p-value	Bonferroni p-value
X1.17_564.3289	-0.92	2.85E-08	4.98E-05	4.98E-05
X8.93_856.6036	1.02	1.65E-06	1.36E-03	2.88E-03
X1.42_566.3497	-2.10	2.34E-06	1.36E-03	4.08E-03
X1.51_592.3513	-22.38	1.92E-05	8.39E-03	3.36E-02
X1.08_612.3280	-3.03	2.50E-05	8.74E-03	4.37E-02
X1.03_562.3132	-8.33	4.10E-05	1.19E-02	
X1.41_452.2762	-6.39	4.85E-05	1.19E-02	
X0.99_586.3136	-4.66	5.80E-05	1.19E-02	
X9.01_1128.5388	7.86	6.13E-05	1.19E-02	
X7.15_786.5626	-1.17	7.19E-05	1.25E-02	
X10.33_871.6904	-8.00	1.19E-04	1.89E-02	
X5.43_1068.6601	-6.19	1.31E-04	1.90E-02	
X1.91_594.3757	-10.83	1.75E-04	2.22E-02	
X5.90_810.5600	-2.99	1.78E-04	2.22E-02	
X8.90_880.6027	1.52	2.36E-04	2.75E-02	
X1.21_476.2768	-2.35	2.60E-04	2.84E-02	
X10.53_854.6676	-2.10	4.62E-04	4.70E-02	
X7.32_864.6056	-4.11	4.89E-04	4.70E-02	
X9.66_768.5494	3.00	5.11E-04	4.70E-02	
X8.61_766.5363	0.65	5.56E-04	4.85E-02	

Supplementary Table 5: Table of lipids significantly associated with serum total adiponectin with FDR p-values <0.05. Beta coefficients, p-values, Benjamini-Hochberg FDR p-values, and Bonferroni p-values listed.

Primary ID	Beta	p-value	FDR p-value	Bonferroni p-value
X1.17_564.3289	0.39	1.14E-07	1.98E-04	1.98E-04
X7.15_786.5626	0.61	1.93E-06	1.31E-03	3.36E-03
X1.42_566.3497	0.93	2.26E-06	1.31E-03	3.94E-03
X4.18_794.5050	3.17	5.94E-06	2.59E-03	1.04E-02
X1.51_592.3513	10.15	1.05E-05	3.66E-03	1.83E-02
X8.62_886.4824	-4.53	3.83E-05	1.11E-02	
X6.50_828.5729	0.18	4.49E-05	1.12E-02	
X5.23_826.5592	0.63	6.07E-05	1.32E-02	
X1.03_562.3132	3.49	1.04E-04	2.01E-02	
X1.21_476.2768	1.09	1.21E-04	2.11E-02	
X8.61_912.4667	-2.37	1.58E-04	2.50E-02	
X4.99_859.5280	1.27	1.94E-04	2.71E-02	
X1.43_702.3160	2.36	2.08E-04	2.71E-02	
X1.49_478.2927	1.48	2.18E-04	2.71E-02	
X1.16_700.3013	1.79	2.50E-04	2.91E-02	
X5.23_1098.4881	1.59	2.73E-04	2.97E-02	
X8.44_716.5227	-1.42	3.36E-04	3.26E-02	
X5.22_1030.5052	2.71	3.52E-04	3.26E-02	
X8.61_980.4565	-2.94	3.55E-04	3.26E-02	
X8.61_896.4927	-2.09	4.81E-04	4.19E-02	
X1.22_544.2648	3.15	5.66E-04	4.70E-02	
X7.47_788.5783	0.73	6.02E-04	4.77E-02	

Supplementary Table 6: Table of lipids significantly associated with serum C-peptide with FDR p-values <0.05. Beta coefficients, p-values, Benjamini-Hochberg FDR p-values, and Bonferroni p-values listed.

Primary ID	Beta	p-value	FDR p-value	Bonferroni p-value
X10.09_880.5267	2.95	8.88E-06	6.25E-03	1.55E-02
X10.09_744.5522	1.04	1.01E-05	6.25E-03	1.76E-02
X9.66_768.5494	2.14	1.07E-05	6.25E-03	1.87E-02
X7.95_790.5381	1.01	5.18E-05	1.68E-02	
X8.44_716.5227	2.05	5.61E-05	1.68E-02	
X8.61_1038.473	3.89	5.76E-05	1.68E-02	
X1.42_566.3497	-1.00	1.10E-04	2.61E-02	
X8.61_766.5363	0.41	1.23E-04	2.61E-02	
X1.17_564.3289	-0.38	1.34E-04	2.61E-02	
X7.15_786.5626	-0.62	2.51E-04	4.37E-02	
X8.93_856.6036	0.45	2.83E-04	4.49E-02	

Supplementary Table 7: Table of primary IDs with Radii >0.1 from singular value decomposition biplot.

Primary ID	Vector 1	Vector 2	Radius
X12.43_934.7918	-0.19	0.13	0.24
X8.17_830.6155	0.03	0.22	0.22
X1.51_592.3513	0.07	0.20	0.22
X12.14_850.7682	0.20	-0.05	0.20
X8.24_880.6145	-0.08	-0.18	0.20
X3.52_421.3076	-0.09	-0.13	0.16
X1.35_669.3989	0.16	0.02	0.16
X10.21_1048.6206	-0.08	0.12	0.15
X3.45_269.1316	0.13	0.00	0.13
X5.27_1031.496	-0.05	0.12	0.13
X12.88_922.7355	0.12	-0.05	0.13
X8.28_814.6742	-0.05	-0.12	0.13
X5.23_964.5107	-0.05	0.11	0.12
X1.57_466.3290	0.12	-0.02	0.12
X13.87_920.717	0.03	-0.11	0.12
X1.2_488.2839	-0.01	0.11	0.12
X9.03_842.5446	0.02	0.11	0.11
X14.35_1062.754	-0.08	-0.08	0.11
X10.38_1017.6967	0.10	0.06	0.11
X9.96_1034.5762	-0.11	0.00	0.11
X0.99_963.3915	-0.10	-0.05	0.11
X1.93_419.3020	0.09	-0.05	0.10
X8.87_1060.5423	-0.10	-0.02	0.10
X1.91_594.3757	0.03	0.10	0.10
X3.52_459.3291	0.08	-0.06	0.10

Supplementary Table 8. Beta coefficients and Bonferroni corrected p-values calculated from the regression of principal component 4 scores (n=126) with 1,745 plasma lipid metabolites.

Positive Betas

Metabolite	Beta	Bonferroni p-value
X13.10_920.7410	3.54	3.63E-05
X8.23_950.6408	3.53	4.02E-04
X13.07_988.7940	3.36	2.29E-02
X9.96_1034.5762	2.46	2.48E-06
X14.31_881.7973	2.38	3.74E-04
X13.11_919.7343	2.26	5.00E-04
X14.38_880.7349	2.19	4.18E-05
X14.33_869.3325	2.05	1.64E-08
X14.35_1062.754	1.99	4.74E-05
X2.65_255.2331	1.98	1.59E-06

Negative Betas

Metabolite	Beta	Bonferroni p-value
X9.03_842.5446	-1.76	6.52E-05
X11.20_1012.7670	-1.25	7.79E-08
X1.91_594.3757	-1.17	1.56E-03
X8.67_864.6048	-1.00	2.63E-02
X10.11_1381.848	-0.92	1.83E-04
X10.33_871.6904	-0.73	4.82E-02
X10.19_802.5695	-0.71	9.17E-08
X4.37_881.5140	-0.69	1.06E-07
X9.45_1261.8069	-0.66	4.26E-04
X0.99_586.3136	-0.65	4.09E-09

Supplementary Table 9: Tables displaying MS/MS spectrum of primary ids (i.e., retention time_*m/z*) generated by Progenesis QI that were significantly associated with responses, but were not listed in lipidomics databases and libraries.

Primary ID: X1.35_669.3989

Measured *m/z* (Da)	% base peak
174.9469	29.32
193.1514	5.68
218.0910	9.22
231.1625	25.90
257.1484	27.50
275.1544	48.08
277.1760	9.09
395.2210	100.00
396.2443	15.57
669.3997	52.81
670.4040	16.69
671.3848	6.04

Primary ID: X4.18_794.5050

Measured *m/z* (Da)	% base peak
540.2896	1.21
568.2869	0.92
794.5059	100.00
795.5094	46.56
796.5105	18.17
797.5401	3.34

Primary ID: X5.43_1068.6601

Measured *m/z* (Da)	% base peak
221.0662	4.70
536.5089	2.45
698.5816	7.19
1022.6674	100.00
1023.6949	61.47
1024.6909	21.31
1025.7070	2.86
1068.7111	3.03

Primary ID: 10.11_1381.8480

Measured *m/z* (Da)	% base peak
755.6339	2.65
1335.8094	100.00
1336.8085	82.18
1337.8382	31.18
1381.8666	8.81
1382.8512	14.69

All MS/MS data were performed on samples with the most abundant parent ion(s) of interest, respectively, using the same chromatography column, solvent gradient, and mass analyzer setting. However, the collision energy used for parent ions MS/MS was increased to a ramp of 20-80V. Spectrum lists display all m/zs with >250 counts, from MS/MS of parent ions.

Supplementary Table 10: Complete list of primary ids, common name, molecular ion, theoretical *m/z*, and mass error of lipids identified.

Primary ID	Common name	Molecular Ion (ESI-neg)	Theoretical *m/z* (Da)	Mass error (Da)
X0.99_586.3136	LPC(20:5)	[M+formate]-	586.3151	0.0015
X1.03_562.3132	LPC(18:3)	[M+formate]-	562.3151	0.0019
X1.08_612.3280	LPC(22:6)	[M+formate]-	612.3307	0.0027
X1.17_564.3289	LPC(18:2)	[M+formate]-	564.3307	0.0018
X1.21_476.2768	LPE(18:2)	[M-H]-	476.2783	0.0015
X1.22_544.2648	LPS(20:4)	[M-H]-	544.2681	0.0033
X1.35_669.3989	Unknown	-		
X1.42_566.3497	LPC(18:1)	[M+formate]-	566.3464	0.0033
X1.49_478.2927	LPE(18:1)	[M-H]-	478.2939	0.0012
X1.51_592.3513	LPC(20:2)	[M+formate]-	592.362	0.0107
X1.59_554.3446	LPC(17:0)	[M+formate]-	554.3464	0.0018
X1.91_594.3757	LPC(20:1)	[M+formate]-	594.3777	0.002
X2.65_255.2331	Palmitic acid	[M-H]-	255.233	0.0001
X3.52_421.3076	TLTI	-		
X4.18_794.5050	Unknown	-		
X4.37_881.5140	PI(16:0_22:6)	[M-H]-	881.5186	0.0046
X5.23_826.5592	PC(18:2_18:2)	[M+formate]-	826.5604	0.0012
X5.43_1068.6601	Unknown	-		
X5.90_810.5600	PC(O-16:0_20:5)	[M+formate]-		
X6.50_828.5729	PC(18:1_18:2)	[M+formate]-	828.576	0.0031
X7.15_786.5626	PC(O-16:1_18:2)	[M+formate]-	786.5655	0.0029
X7.32_864.6056	PC(O-18:1_22:5)	[M+formate]-		
X7.47_788.5783	PC(O-16:0_18:2)	[M+formate]-		
X8.17_830.6155	PC(18:0_18:2)	[M+formate]-	830.5917	0.0238
X8.23_950.6408	TLTI	-		
X8.24_880.6145	PC(20:1_20:4)	[M+formate]-		
X8.44_716.5227	PE(16:0_18:1)	[M-H]-	716.5236	0.0009
X8.61_1038.473	TLTI	-		
X8.61_766.5363	PE(18:0_20:4)	[M-H]-	766.5392	0.0029
X8.67_864.6048	PC(O-18:0_22:6)	[M+formate]-	864.6124	0.0076
X8.90_880.6027	PC(18:0_22:5)	[M+formate]-		
X8.93_856.6036	PC(18:0_20:3)	[M+formate]-	856.6073	0.0037
X9.01_1128.5388	TLTI	-		
X9.03_842.5446	TLTI	-		
X9.42_814.5927	PC(O-18:1_18:2)	[M+formate]-	814.5968	0.0041
X9.45_1261.8069	TLTI	-		
X9.66_768.5494	PE(18:0_20:3)	[M-H]-	768.5549	0.0055
X9.96_1034.5762	TLTI	-		
X10.11_1381.8480	Unknown	-		
X10.19_802.5695	TLTI	-		

X10.33_871.6904	SM(d19:1/24:1)	[M+formate]$^-$	871.691	0.0006
X11.20_1012.7670	TLTI	-		
X12.14_850.7682	TLTI	-		
X12.43_934.7918	TLTI	-		
X13.07_988.7940	TLTI	-		
X13.10_920.7410	TLTI	-		
X13.11_919.7343	TLTI	-		
X14.31_881.7973	TLTI	-		
X14.33_869.3325	TLTI	-		
X14.35_1062.754	TLTI	-		
X14.38_880.7349	TLTI	-		

Supplementary Table 11: Relationship between untargeted PL FA chains and the % of geometric and positional FA isomers in plasma PL

Common name[a]	Palmitic[b] C16:0	Stearic[b] C18:0	Oleic[b] c-C18:1	Elaidic[b] t-C18:1	Eicosenoic[b] C20:1	Linoleic[b] c-C18:2	Linoelaidic[b] t-C18:2	ALA[b] C18:3	Eicosadienoic[b] C20:2	DGLA[b] C20:3	ARA[b] C20:4	EPA[b] C20:5	DPA ω-3[b] C22:5	DPAω-6[b] C22:5	DHA[b] C22:6	Nervonic[b] C24:1
LPC(20:5)	-	-	-	-	-	-	-	-	-	-	-	**0.61**	-	-	-	-
LPC(18:3)	-	-	-	-	-	-	-	**0.28**	-	-	-	-	-	-	-	-
LPC(22:6)	-	-	-	-	-	-	-	-	-	-	-	-	-	-	**0.60**	-
LPC(18:2)	-	-	-	-	-	**0.55**	-0.03	-	-	-	-	-	-	-	-	-
LPE(18:2)	-	-	-	-	-	**0.53**	-0.01	-	-	-	-	-	-	-	-	-
LPS(20:4)	-	-	-	-	-	-	-	-	-	-	**-0.39**	-	-	-	-	-
LPC(18:1)	-	-	**0.21**	0.02	-	-	-	-	-	-	-	-	-	-	-	-
LPE(18:1)	-	-	**0.19**	0.06	-	-	-	-	-	-	-	-	-	-	-	-
LPC(20:2)	-	-	-	-	-	-	-	-	**0.18**	-	-	-	-	-	-	-
LPC(17:0)	-	-	-	-	-	-	-	-	-	-	-	-	-	-	-	-
LPC(20:1)	-	-	-	-	0.16	-	-	-	-	-	-	-	-	-	-	-
Palmitic acid	**0.23**	-	-	-	-	-	-	-	-	-	-	-	-	-	-	-
PI(16:0_22:6)	-0.03	-	-	-	-	-	-	-	-	-	-	-	-	-	**0.45**	-
PC(18:2_18:2)	-	-	-	-	-	**0.67**	**-0.20**	-	-	-	-	-	-	-	-	-
PC(O-16:0_20:5)	-	-	-	-	-	-	-	-	-	-	-	**0.48**	-	-	-	-
PC(18:1_18:2)	-	-	0.06	-0.10	-	**0.53**	-0.17	-	-	-	-	-	-	-	-	-
PC(O-16:1_18:2)	-	-	-	-	-	**0.45**	-0.15	-	-	-	-	-	-	-	-	-
PC(O-18:1_22:5)	-	-	-	-	-	-	-	-	-	-	-	-	**0.24**	-0.05	-	-
PC(O-16:0_18:2)	-	-	-	-	-	**0.46**	-0.15	-	-	-	-	-	-	-	-	-
PC(18:0_18:2)	-	0.06	-	-	-	**0.26**	**-0.18**	-	-	-	-	-	-	-	-	-
PC(20:1_20:4)	-	-	-	-	0.16	-	-	-	-	-	0.02	-	-	-	-	-
PE(16:0_18:1)	**0.34**	-	**0.20**	**-0.04**	-	-	-	-	-	-	-	-	-	-	-	-
PE(18:0_20:4)	-	**0.28**	-	-	-	-	-	-	-	-	0.12	-	-	-	-	-
PC(O-18:0_22:6)	-	-	-	-	-	-	-	-	-	-	-	-	-	-	**0.43**	-
PC(18:0_22:5)	-	**0.40**	-	-	-	-	-	-	-	-	-	-	-0.15	**0.56**	-	-
PC(18:0_20:3)	-	**0.28**	-	-	-	-	-	-	-	**0.52**	-	-	-	-	-	-
PC(O-18:1_18:2)	-	-	-	-	-	**0.27**	-0.12	-	-	-	-	-	-	-	-	-
PE(18:0_20:3)	-	**0.30**	-	-	-	-	-	-	-	**0.34**	-	-	-	-	-	-
SM(d19:1/24:1)	-	-	-	-	-	-	-	-	-	-	-	-	-	-	-	**0.37**

Spearman correlations were performed to determine the relationship between the abundance of each plasma PL significantly associated with the responses in our study and the % of FA isomers in plasma PL from our previous study. For instance, LPC(18:2)

and LPE(18:2) were significantly correlated with the % of LA (ω-6, $C18:2^{\Delta 9Z,12Z}$), whereas LPC(18:2) and LPE(18:2) were not correlated with the % of trans-isomer linoelaidic acid (ω-6, $C18:2^{\Delta 9E,12E}$). Numeric values represent the Spearman correlation coefficients and are bolded if $p<0.05$. The plasma analyzed from both data sets were collected at the same time point from the same patients.

[a] The significant PL associated with the responses that were structurally characterized in our study. The experimental methodology employed was the UPLC-ESI-MS^E analysis of crude lipid extracts outlined in this manuscript

[b] The geometric and positional FA isomers that were targeted in our previous study (Pickens et al. PLEFA. 2015.). The experimental methodology employed was the FAME analysis of isolated plasma PL by GC-FI

Supplementary Table 12: R code used in statistical analyses presented in the manuscript.

```
############################
######  Loading Data  ########
############################

load("human.data.rda") # Object H. Identifier data of 126 study participants, includes: age, smoking, BMI, WC,
 # serum markers
dim(H) # 126 rows
load("IS mean imputed.rda") # Data matrix of internal standard normalized mean imputed lipidomic data
dim(IS.mean) # 126 rows and 1745 lipids
all.equal.character(rownames(H), rownames(IS.mean)) #The identifier data for each patient is aligned with their
# lipidomic data

##############################
######  Scaling of Data  ########
##############################

#### Pareto Scaling of Internal Standard Normalize Mean imputed Data  #####
 install.packages("MetabolAnalyze")
 library(MetabolAnalyze) # Package used for pareto scaling.
    # https://cran.r-project.org/web/packages/MetabolAnalyze/index.html
 pareto.IS = scaling(as.matrix(scale(IS.mean, center=T, scale=F)), type = "pareto") # Center and pareto scale the
 internal standard normalized mean imputed data.
 dim(pareto.IS) # 126 rows and 1745 lipids
 all.equal.character(rownames(H), rownames(pareto.IS)) # The identifier data for each patient is aligned with their
 # scaled lipidomic data


####################################
###### ANOVA and Post Hoc  #########
####################################

#### Kruskal Wallis (KW) one-way ANOVA was conducted across BMI categories along
 # with Dunn's test for multiple comparison  #####

### KW one-way ANOVA ###
 kwpv=numeric() # Numeric vector for indexing KW ANOVA p-values
 ## Loop for KW ANOVA of age, BMI, WC, and serum adipokines, cytokines, and C-peptide
 # across BMI category

 for (i in 1:ncol(H)){
   kwpv[i]=kruskal.test(x=H[,i], g=H$BMI.cat)$p.value # Indexing the p-value from each ANOVA
 }

 kwbh=p.adjust(kwpv, method = "BH") # Adjusting KW p-values according to Benjamini-Hochberg

### Dunn non-parametric test for multiple comparison ###
library(PMCMR)
 tmp=matrix(NA, ncol = 4, nrow = ncol(H))
 colnames(tmp)=c("var","lean-overwt","lean-obese", "overwt-obese" )
 tmp[,1]=colnames(H)

for (i in 1:ncol(H)){
  tmpindex=posthoc.kruskal.dunn.test(x=H[,i], g=H$BMI.cat, p.adjust.method = "BH")
  tmpgetp=get.pvalues(tmpindex)
```

```
  tmp[i,2]=tmpgetp[[1]]
  tmp[i,3]=tmpgetp[[2]]
  tmp[i,4]=tmpgetp[[3]]
}

########################################
######  Bayesian Analysis of Traits  ########
########################################

#### Reproducing Kernel Hilbert Spaces (RKHS) Regressions ####
 install.packages("BGLR")
 library(BGLR)
  # For more information on using BGLR refer to https://github.com/gdlc/BGLR-R

 ### Creating a directory to store file outputs from BGLR ###
 dir.create("BMI"); setwd("BMI")
 getwd() # Verify working directory

 ### Setting response, lipidomic data, iterations, and burn in  ###
 X=pareto.IS # 1,745 pareto scaled lipidomic data
 y<-H$BMI # Response: body mass index values for each respective patient
 nIter=200000 # Long Markov Chain of 200,000 iterations
 burnIn=50000 # Number of iterations to discard for burn in

    ## Computing the metabolomic similarity matrix ##
    L<-sum(apply(X=X,FUN=var,MARGIN=2))
    G<-tcrossprod(X)/L
     # The G matrix is an nxn matrix of distances to measure similarities
       # between participants with respect to their lipid profiles

    ##  RKHS model parameters  ##
     # ETA #
     ETA.FixMet=list(Met=list(K=G,model="RKHS"), # The G matrix represents the lipidome,
                    # and the RKHS kernel is specified
       Fix=list(~H$age+factor(H$smoking), model="FIXED")) # The fixed effects of the model are age and smoking

    ## RKHS regression ##
    fmGBLUP<-BGLR(y=y,ETA=ETA.FixMet, nIter=nIter, burnIn=burnIn, saveAt="GBLUP_")
       # RKHS model is: BMI = fixed effects + lipidomic data

 ### Variance of Best Linear Unbiased Predictor (BLUP) and variance of error ###
   # inference was done based on one of every 5 samples of the last 150,000
    # therefore, since the burn in was 50,000 we need to remove the first 10,000 samples
 list.files()
 VarU=scan("GBLUP_ETA_Met_varU.dat") #load in variance of the lipidome BLUP model (varU). Total of 30,000
          # lines.
 VarU=VarU[-c(1:10000)] # remove the burn in iterations from varU
 VarE=scan("GBLUP_varE.dat") # load in variance of the error (varE). Total of 30,000 lines.
 VarE=VarE[-c(1:10000)] #remove the burn in iterations varE

   ## Calculating percent of the inter-individual differences in response variables that can be
     # attributed to lipidome profiles ##
    tmp=(VarU/(VarU+VarE))*100 # Calculate % varU as sum of varU + varE
    round(quantile(tmp, probs = c(0.025, 0.975)),0) # Determine 95% confidence intervals

###### The other responses were run similarly by creating individual directories for each response and modifying
```

Supplementary Information

```
 # the y<- for each individual response  #######


#################################
#####  Single Lipid Regressions #####
#################################

### Setting the model response and independent variables ###
 X=pareto.IS # Data set
 XX=H$BMI # Response, dependent variable

### Creating numeric vectors for indexing estimated effect and model p-value ###
 BMI.mypv=numeric(ncol(X)) # Numeric variable for indexing each p-value for each lipid
 BMI.myB=numeric(ncol(X)) #Beta coefficient for each lipid

### Loop of single marker regressions of pareto scaled data ###
 for (i in 1:ncol(X)){ # For each column of data matrix
  tmp=summary(lm(XX~ H$age + H$smoking + X[,i])) # list of summary of each linear model
        BMI.mypv[i]=tmp$coefficients[5,4]  # Indexing p-value
        BMI.myB[i]=tmp$coefficients[5,1]  # Indexing beta coefficent
 }

###  P-value adjustments  ###
 ##  Bonferroni pvalue correction  ##
        p.bon=p.adjust(BMI.mypv, method='bonferroni')

 ## Benjamini-Hochberg pvalue correction ##
        p.BH=p.adjust(BMI.mypv, method='BH')

### Creating a dataframe of regression results ###
      Complete.Regression.Report=data.frame('Metabolite'=colnames(X), BMI.stats.reportBMI.myB, BMI.mypv, 'BMI.BH'=
 p.BH,  'BMI.bon'=p.bon) # Compile all Response beta coefficients, p-values, and corrected p-values

###### The other responses were run similarly by modifying the response input (i.e., either BMI, WC, leptin, etc)
# and uniquely indexing model outputs and compiling their results into the file Complete.Regression.Report  ######


#######################################
##### Singular Value Decomposition  #####
#######################################
load("beta.report.rda")
# Beta.Report is a dataframe with 1745 rows and 10 columns
 # Column 1 contains the name of each of the 1745 lipids
  # Columns 2-10 contain the 1745 estimated effects for each respective response (i.e., BMI, WC,
      # leptin, etc)

# Singular value decompositions (SVD) is defined as
 # A= U*D*V
  # where U is an mxm matrix, D is an mxn, and V is an nxn matrix

### Singular value decomposition of traits ###
pca.beta=princomp(Beta.Report[,2:ncol(Beta.Report)])
lambda=pca.beta$sdev*sqrt(pca.beta$n.obs)
score=t(t(pca.beta$scores)/lambda)
variables=t(t(pca.beta$loadings)*lambda)
```

Supplementary Information

```
v.1=variables
v.1[8,1]=v.1[8,1]/25 #scaling MCP-1 Pc1 score by a factor of 25


#############################################################
###############  Principal Components Analysis ################
#############################################################

#### Setting response and computing principal components (PCs) ####
  XX=H$BMI # Response, dependent variable to be used in regressions
  Z=tcrossprod(pareto.IS) # Computing an nxn matrix of lipidomic data
  ZZ=eigen(Z) # Computing Eigen values and Eigen vectors of nxn matrix of lipidomic data
  EV=(ZZ$values/sum(ZZ$values))*100 # Calculating the Eigen value % of total variation


### Creating vectors to index pvalue and beta coefficient
 PCA.mypv=numeric(ncol(Z[,1:125])) #p-value for each metabolite
 PCA.myB=numeric(ncol(Z[,1:125])) #Beta coefficent for each metabolite

### Loop of BMI regressed on each PC individually ###
for (i in 1:125){ # For each PC
  tmp=summary(lm(XX~ H$age + H$smoking + ZZ$vectors[,i])) # List of outputs from linear model
  PCA.mypv[i]=tmp$coefficients[4,4] # Indexing the p-value from linear model
  PCA.myB[i]=tmp$coefficients[4,1] # Indexing the beta coefficients from linear model
}

###  P-value adjustments  ###
  ##  Bonferroni pvalue correction  ##
    PCA.pbon=p.adjust(PCA.mypv, method='bonferroni')


  ## Benjamini-Hochberg pvalue correction ##
    PCA.pBH=p.adjust(PCA.mypv, method='BH')


#######################################################
######### Determining lipids driving PC4 loadings  #########
#######################################################

#### Setting model response and creating vectors for indexing  ####
  Y=ZZ$vectors[,4] # Response, dependent variable is PC4 loadings
  b=numeric() # Numeric vector for indexing beta coefficients
  pval=numeric() # Numeric vector for indexing p-values

  ###  Loop of PC4 scores regressed on each lipid individually  ###
    for (i in 1:ncol(pareto.IS)){ # For each of the 1745 lipids
      tmp=summary(lm(Y~pareto.IS[,i])) # List of summary of each linear model
      b[i]=tmp$coefficients[2,1] # Indexing beta coefficients
      pval[i]=tmp$coefficients[2,4] # Indexing p-values
    }

  ###  P-value adjustments  ###
    ##  Bonferroni pvalue correction  ##
      p.bon=p.adjust(pval, method='bonferroni')

    ## Benjamini-Hochberg pvalue correction ##
```

```
p.BH=p.adjust(pval, method='BH')

####################################################################
######### Phospholipid FAME data and untargeted lipid associations #########
####################################################################

all.equal(rownames(H),rownames(pareto.IS))

X=data.frame(pareto.IS)

#           X0.99_586.3136  LPC(20:5)
cor.test(X$X0.99_586.3136, H$EPA, method="spearman")
summary(lm(log(X$X0.99_586.3136)~log(H$EPA)+ H$BMI ))

#           X1.03_562.3132  LPC(18:3)
cor.test(X$X1.03_562.3132, H$ALinolenic, method="spearman")
summary(lm(log(X$X1.03_562.3132)~ log(H$ALinolenic) +H$BMI))

#           X1.08_612.3280  LPC(22:6)
cor.test(X$X1.08_612.328, H$DHA, method="spearman")
summary(lm(log(X$X1.08_612.328)~ log(H$DHA) +H$BMI))


#           X1.17_564.3289  LPC(18:2)
cor.test(X$X1.17_564.3289, H$Linoleic, method="spearman")
cor.test(X$X1.17_564.3289, H$Linoelaidic, method="spearman")
summary(lm(log(X$X1.17_564.3289)~ log(H$Linoleic) +H$BMI))
summary(lm(log(X$X1.17_564.3289)~ log(H$Linoelaidic) +H$BMI))


#           X1.21_476.2768  LPE(18:2)
cor.test(X$X1.21_476.2768, H$Linoleic, method="spearman")
cor.test(X$X1.21_476.2768, H$Linoelaidic, method="spearman")
summary(lm(log(X$X1.21_476.2768)~ log(H$Linoleic) +H$BMI))
summary(lm(log(X$X1.21_476.2768)~ log(H$Linoelaidic) +H$BMI))

#           X1.22_544.2648  LPS(20:4)
cor.test(X$X1.22_544.2648, H$AA, method="spearman")
summary(lm(log(X$X1.22_544.2648)~ log(H$AA) +H$BMI))

#           X1.42_566.3497  LPC(18:1)
cor.test(X$X1.42_566.3497, H$Oleic, method="spearman")
cor.test(X$X1.42_566.3497, H$Elaidic, method="spearman")
summary(lm(log(X$X1.42_566.3497)~ log(H$Oleic) +H$BMI))
summary(lm(log(X$X1.42_566.3497)~ log(H$Elaidic) +H$BMI))

#           X1.49_478.2927  LPE(18:1)
cor.test(X$X1.49_478.2927, H$Oleic, method="spearman")
cor.test(X$X1.49_478.2927, H$Elaidic, method="spearman")
summary(lm(log(X$X1.49_478.2927)~ log(H$Oleic) +H$BMI))
summary(lm(log(X$X1.49_478.2927)~ log(H$Elaidic) +H$BMI))

#           X1.51_592.3513  LPC(20:2)
cor.test(X$X1.51_592.3513, H$Eicosadienoic, method="spearman")
summary(lm(log(X$X1.51_592.3513)~ log(H$Eicosadienoic) +H$BMI))
```

Supplementary Information

```
#           X1.59_554.3446  LPC(17:0)
  # We didn't target this

#           X1.91_594.3757  LPC(20:1)
cor.test(X$X1.91_594.3757, H$Eicosenoic, method="spearman")
summary(lm(log(X$X1.91_594.3757)~ log(H$Eicosenoic) +H$BMI))

#           X2.65_255.2331  Palmitic acid
cor.test(X$X2.65_255.2331, H$Palmitic, method="spearman")
summary(lm(log(X$X2.65_255.2331)~ log(H$Palmitic) +H$BMI))

#           X4.37_881.5140  PI(16:0_22:6)
cor.test(X$X4.37_881.514, H$Palmitic, method="spearman")
cor.test(X$X4.37_881.514, H$DHA, method="spearman")
summary(lm(log(X$X4.37_881.514)~ log(H$Palmitic) +H$BMI))
summary(lm(log(X$X4.37_881.514)~ log(H$DHA) +H$BMI))

#           X5.23_826.5592  PC(18:2_18:2)
cor.test(X$X5.23_826.5592, H$Linoleic, method="spearman")
cor.test(X$X5.23_826.5592, H$Linoelaidic, method="spearman")
summary(lm(log(X$X5.23_826.5592)~ log(H$Linoleic) +H$BMI))
summary(lm(log(X$X5.23_826.5592)~ log(H$Linoelaidic) +H$BMI))

#           X5.90_810.5600  PC(O-16:0_20:5)
cor.test(X$X5.9_810.56, H$EPA, method="spearman")
summary(lm(log(X$X5.9_810.56)~ log(H$EPA) +H$BMI))

#           X6.50_828.5729  PC(18:1_18:2)
cor.test(X$X6.5_828.5729, H$Oleic, method="spearman")
cor.test(X$X6.5_828.5729, H$Elaidic, method="spearman")
cor.test(X$X6.5_828.5729, H$Linoleic, method="spearman")
cor.test(X$X6.5_828.5729, H$Linoelaidic, method="spearman")
summary(lm(log(X$X6.5_828.5729)~ log(H$Oleic) +H$BMI))
summary(lm(log(X$X6.5_828.5729)~ log(H$Elaidic) +H$BMI))
summary(lm(log(X$X6.5_828.5729)~ log(H$Linoleic) +H$BMI))
summary(lm(log(X$X6.5_828.5729)~ log(H$Linoelaidic) +H$BMI))

#           X7.15_786.5626  PC(O-16:1_18:2)
cor.test(X$X7.15_786.5626, H$Linoleic, method="spearman")
cor.test(X$X7.15_786.5626, H$Linoelaidic, method="spearman")
summary(lm(log(X$X7.15_786.5626)~ log(H$Linoleic) +H$BMI))
summary(lm(log(X$X7.15_786.5626)~ log(H$Linoelaidic) +H$BMI))

#           X7.32_864.6056  PC(O-18:1_22:5)
cor.test(X$X7.32_864.6056, H$DPA3, method="spearman")
cor.test(X$X7.32_864.6056, H$DPA6, method="spearman")
summary(lm(log(X$X7.32_864.6056)~ log(H$DPA3) +H$BMI))
summary(lm(log(X$X7.32_864.6056)~ log(H$DPA6) +H$BMI))

#           X7.47_788.5783  PC(O-16:0_18:2)
cor.test(X$X7.47_788.5783, H$Linoleic, method="spearman")
cor.test(X$X7.47_788.5783, H$Linoelaidic, method="spearman")
summary(lm(log(X$X7.47_788.5783)~ log(H$Linoleic) +H$BMI))
summary(lm(log(X$X7.47_788.5783)~ log(H$Linoelaidic) +H$BMI))

#           X8.17_830.6155  PC(18:0_18:2)
```

Supplementary Information

```
cor.test(X$X8.17_830.6155, H$Stearic, method="spearman")
cor.test(X$X8.17_830.6155, H$Linoleic, method="spearman")
cor.test(X$X8.17_830.6155, H$Linoelaidic, method="spearman")
summary(lm(log(X$X8.17_830.6155)~ log(H$Stearic) +H$BMI))
summary(lm(log(X$X8.17_830.6155)~ log(H$Linoleic) +H$BMI))
summary(lm(log(X$X8.17_830.6155)~ log(H$Linoelaidic) +H$BMI))

#	X8.24_880.6145  PC(20:1_20:4)
cor.test(X$X8.24_880.6145, H$Eicosenoic, method="spearman")
cor.test(X$X8.24_880.6145, H$AA, method="spearman")
summary(lm(log(X$X8.24_880.6145)~ log(H$Eicosenoic) +H$BMI))
summary(lm(log(X$X8.24_880.6145)~ log(H$AA) +H$BMI))

#	X8.44_716.5227  PE(16:0_18:1)
cor.test(X$X8.44_716.5227, H$Palmitic, method="spearman")
cor.test(X$X8.44_716.5227, H$Oleic, method="spearman")
cor.test(X$X8.44_716.5227, H$Elaidic, method="spearman")
summary(lm(log(X$X8.44_716.5227)~ log(H$Palmitic) +H$BMI))
summary(lm(log(X$X8.44_716.5227)~ log(H$Oleic) +H$BMI))
summary(lm(log(X$X8.44_716.5227)~ log(H$Elaidic) +H$BMI))

#	X8.61_766.5363  PE(18:0_20:4)
cor.test(X$X8.61_766.5363, H$Stearic, method="spearman")
cor.test(X$X8.61_766.5363, H$AA, method="spearman")
summary(lm(log(X$X8.61_766.5363)~ log(H$Stearic) +H$BMI))
summary(lm(log(X$X8.61_766.5363)~ log(H$AA) +H$BMI))

#	X8.67_864.6048  PC(O-18:0_22:6)
cor.test(X$X8.67_864.6048, H$DHA, method="spearman")
summary(lm(log(X$X8.67_864.6048)~ log(H$DHA) +H$BMI))

#	X8.90_880.6027  PC(18:0_22:5)
cor.test(X$X8.9_880.6027, H$Stearic, method="spearman")
cor.test(X$X8.9_880.6027, H$DPA3, method="spearman")
cor.test(X$X8.9_880.6027, H$DPA6, method="spearman")
summary(lm(log(X$X8.9_880.6027)~ log(H$Stearic) +H$BMI))
summary(lm(log(X$X8.9_880.6027)~ log(H$DPA3) +H$BMI))
summary(lm(log(X$X8.9_880.6027)~ log(H$DPA6) +H$BMI))

#	X8.93_856.6036  PC(18:0_20:3)
cor.test(X$X8.93_856.6036, H$Stearic, method="spearman")
cor.test(X$X8.93_856.6036, H$DGLA, method="spearman")
summary(lm(log(X$X8.93_856.6036)~ log(H$Stearic) +H$BMI))
summary(lm(log(X$X8.93_856.6036)~ log(H$DGLA) +H$BMI))

#	X9.42_814.5927  PC(O-18:1_18:2)
cor.test(X$X9.42_814.5927, H$Linoelaidic, method="spearman")
cor.test(X$X9.42_814.5927, H$Linoleic, method="spearman")
summary(lm(log(X$X9.42_814.5927)~ log(H$Linoleic) +H$BMI))
summary(lm(log(X$X9.42_814.5927)~ log(H$Linoelaidic) +H$BMI))

#	X9.66_768.5494  PE(18:0_20:3)
cor.test(X$X9.66_768.5494, H$Stearic, method="spearman")
cor.test(X$X9.66_768.5494, H$DGLA, method="spearman")
summary(lm(log(X$X9.66_768.5494)~ log(H$Stearic) +H$BMI))
summary(lm(log(X$X9.66_768.5494)~ log(H$DGLA) +H$BMI))
```

Supplementary Information

```
#            X10.33_871.6904              SM(d19:1/24:1)
cor.test(X$X10.33_871.6904, H$Nervonic, method="spearman")
summary(lm(log(X$X10.33_871.6904)~ log(H$Nervonic) +H$BMI))
```
